# Influence of Ce^3+^ Substitution on Antimicrobial and Antibiofilm Properties of ZnCe_x_Fe_2−x_O_4_ Nanoparticles (X = 0.0, 0.02, 0.04, 0.06, and 0.08) Conjugated with Ebselen and Its Role Subsidised with γ-Radiation in Mitigating Human TNBC and Colorectal Adenocarcinoma Proliferation In Vitro

**DOI:** 10.3390/ijms221810171

**Published:** 2021-09-21

**Authors:** Mohamed K. Abdel-Rafei, Noura M. Thabet, M. I. A. Abdel Maksoud, M. Abd Elkodous, Go Kawamura, Atsunori Matsuda, A. H. Ashour, Ahmed I. El-Batal, Gharieb S. El-Sayyad

**Affiliations:** 1 Radiation Biology Department, National Center for Radiation Research and Technology (NCRRT), Egyptian Atomic Energy Authority (EAEA), Cairo 11787, Egypt; mohamed.marawan2011@yahoo.com; 2 Materials Science Lab., Radiation Physics Department, National Center for Radiation Research and Technology (NCRRT), Egyptian Atomic Energy Authority (EAEA), Cairo 11787, Egypt; muhamadmqsod@gmail.com (M.I.A.A.M.); ashourvip3@hotmail.com (A.H.A.); 3 Department of Electrical and Electronic Information Engineering, Toyohashi University of Technology, 1-1 Hibarigaoka, Tempaku-cho, Toyohashi 441-8580, Aichi, Japan; mohamed.hamada.abdlekodous.xi@tut.jp (M.A.E.); kawamura.go.km@tut.jp (G.K.); 4 Drug Microbiology Lab., Drug Radiation Research Department, National Center for Radiation Research and Technology (NCRRT), Egyptian Atomic Energy Authority (EAEA), Cairo 11787, Egypt; aelbatal2020@gmail.com (A.I.E.-B.); Gharieb.S.Elsayyad@eaea.org.eg (G.S.E.-S.)

**Keywords:** MDA-MB-231, HT-29, ebselen, cerium, ERK1/2, STAT-6, IL-4, STAT-1, antimicrobial activity

## Abstract

Cancers are a major challenge to health worldwide. Spinel ferrites have attracted attention due to their broad theranostic applications. This study aimed at investigating the antimicrobial, antibiofilm, and anticancer activities of ebselen (Eb) and cerium-nanoparticles (Ce-NPs) in the form of ZnCe_x_Fe_2−X_O_4_ on human breast and colon cancer cell lines. Bioassays of the cytotoxic concentrations of Eb and ZnCe_x_Fe_2−X_O_4_, oxidative stress and inflammatory milieu, autophagy, apoptosis, related signalling effectors, the distribution of cells through the cell-cycle phases, and the percentage of cells with apoptosis were evaluated in cancer cell lines. Additionally, the antimicrobial and antibiofilm potential have been investigated against different pathogenic microbes. The ZOI, and MIC results indicated that ZnCe_x_Fe_2−X_O_4_; X = 0.06 specimen reduced the activity of a wide range of bacteria and unicellular fungi at low concentration including *P. aeruginosa *(9.5 mm; 6.250 µg/mL), *S. aureus* (13.2 mm; 0.390 µg/mL), and *Candida albicans* (13.5 mm; 0.195 µg/mL). Reaction mechanism determination indicated that after ZnCe_x_Fe_2−x_O_4_; X = 0.06 treatment, morphological differences in *S.* *aureus* were apparent with complete lysis of bacterial cells, a concomitant decrease in the viable number, and the growth of biofilm was inhibited. The combination of Eb with ZFO or ZnCe_x_Fe_2−X_O_4_ with γ-radiation exposure showed marked anti-proliferative efficacy in both cell lines, through modulating the oxidant/antioxidant machinery imbalance, restoring the fine-tuning of redox status, and promoting an anti-inflammatory milieu to prevent cancer progression, which may be a valuable therapeutic approach to cancer therapy and as a promising antimicrobial agent to reduce the pathogenic potential of the invading microbes.

## 1. Introduction

Spinel ferrite is a class of magnetic materials that derives its name from its similarity to the naturally occurring mineral. Spinel ferrites have possible application in areas such as water treatment, data storage, the segregation of biomolecules, colour imaging, therapeutic diagnosis, antimicrobial activities, cores of transformers, bubble devices, electronic communication devices, sensors, and drug delivery [1,2,3,4]. Structure studies of spinels showed that the size of the cations in a sample plays a vital role in determining their site occupancy preferences. The presence of larger ions shifts the oxygen ions diagonally and expands the lattice parameter. The distribution of cations over the sub-lattices has a significant effect on both the chemical and physical properties of the spinel structure and subsequently affects their applications and performance [5,6]. Manipulation of the physical properties of Co-Zn spinel ferrite nanoparticles (NPs) by the incorporation of larger ions into their structure has attracted the attention of researchers. For example, Pawar et al. [7] have addressed the changes induced in the optical properties of cobalt–zinc ferrite Co_0.7_Zn_0.3_Ho_x_Fe_2−x_O_4_ (0 ≤ x ≤ 0.1) due to the insertion of (Ho^3+^), using a facile sol–gel method. These researchers found that the energy bandgap rose from 1.72 to 1.84 eV when the x increased from 0.0 to 0.1. Panda et al. [8] have described the consequences of the incorporation of chromium (Cr^3+^ ( ions into the system CoFe_2−x_Cr_x_O_4_ (X = 0, 0.15, 0.3). They found that the coercivity was lowered upon the insertion of Cr^3+^ due to the magnetic coupling with a weaker magnetic moment of Cr^3+^ (3µ_B_). Farid et al. [9] substituted praseodymium (Pr^3+^) instead of Fe^3+^ into the system Co_0.6_Zn_0.4_Pr_x_Fe_2__−__x_O_4_ (X = 0.0, 0.025, 0.05, 0.075, 0.10). They found that the insertion of Pr^3+^ increased the lattice constant due to the large difference between the ionic radii of Pr^3+^ and Fe^3+^. The resistivity and activation energy also increased with the Pr^3+^ substitution. In our previous work, we synthesised Co-Zn spinel ferrites (ZCFO) NPs via a sol–gel method. The ZCFO sample showed a low crystallite size (11.7872 nm) and high surface area (106.63 m^2^.g), which made it suitable for environmental applications. ZCFO NPs have been used as antimicrobial agents [10], biosensors [11], and as a promising catalyst [12].

Colon cancer is the third most common cancer globally and is the second most common cause of cancer mortality after lung cancer. Approximately 5% of colon cancer patients have an additional primary cancer. In comparison, breast cancer is the single most common cancer of women worldwide, is responsible for 30% of all cancer diagnoses in women, and has a mortality rate of approximately 14% in women. About 3% of breast cancer patients have an additional primary cancer. Breast cancer susceptibility genes have been found to increase the susceptibility to colon cancer of patients with breast cancer [13].

The tumour microenvironment (TME) is a complex network composed of multipotent cells such as stromal cells, mesenchymal stem cells, fibroblasts, blood vessels, endothelial cell precursors and immune cells, and secreted mediators such as cytokines, growth factors, and reactive oxygen species (ROS), which are related to the initiation and maintenance of tumorigenesis. Oncogenic pathways can be associated with major changes in the TME to induce proliferation and inhibit apoptosis and promote angiogenesis and avoid hypoxia as well as inhibit the immune detection and activate immune cells to support invasion and metastasis [14]. Hence, cancer manipulation targeting the components of the TME including metabolites, ROS, hypoxia, and cytokine-mediated inflammation could be a valuable approach to cancer therapy. In radio-therapeutic oncology, radiation directly and indirectly, via radiolysis, induces damage to the structure of DNA, proteins, and lipids, leading to injury of the organelles and cell membranes of cancer cells. Irradiation also disrupts the immunogenicity and microenvironment of cancer cells. These factors have a vital role in regulating cancer cell mitosis, apoptosis, necrosis, proliferation, and other biological functions [15]. Radiotherapy is considered to be an effective treatment option after surgery. However, colorectal cancer exhibits resistance to ionising radiation (IR) as used in radiation oncology treatment. The high doses of radiation required to be delivered to a tumour also result in damage to adjacent normal tissues or organs [16]. Triple-negative breast cancer (TNBC) is the most aggressive breast cancer subtype and has a poor prognosis. Radiotherapy plays an important role in treating TNBC [17]. Hence, there is an urgent need for the development of drugs that serve as radiosensitisers to overcome the radioresistance exhibited by various cancers, including colorectal and TNBC [16,18].

In the current study, we supplemented radiotherapy with theranostic nanoparticles constructed using several approaches such as by conjugating therapeutic agents to imaging nanoparticles, then imaging agents to therapeutic nanoparticles, and hence engineering unique nanoparticles possessing both therapeutic and diagnostic abilities [19]. Ebselen (Eb) (*N*-phenyl-1,2-benzisoselenazol-3(2H)-one) is one of the organo-selenium compounds that mimics glutathione peroxidase and exhibits a wide range of biological activities including antibacterial, cyto-protective, anti-inflammatory, antioxidant, and anticancer activities [20]. In biomedical applications, nanoparticles have attracted attention due to their physiochemical properties such as appropriate size, large surface-to-mass ratios, high reactivity, and the ability to modify the biological influence of diffusivity and immunogenicity [21]. The rare earth element cerium (Ce), which has the electronic configuration [Xe] 4 f26s2, has valuable properties due to its possession of shielded 4f electrons. Ce can exist in two common oxidation states, Ce^3+^ and Ce^4+^ [22]. In biology, cerium oxide nanoparticles (Ce-NPs) have attracted particular interest because of their regenerative and multi-enzymatic scavenging of ROS. The unique antioxidant/catalytic properties of Ce-NPs stem from their reversible switching between the oxidation states of Ce^3+^ and Ce^4+^ and their low reduction potential of around 1.52 V23. The bulk of crystals of cerium dioxide consists of Ce^4+^, but the nano-dimensions reduce the size to Ce^3+^, resulting in a higher activity of biological antioxidant processes [19,22]. We synthesised the ZnFeO_4_ (ZFO) system using the sol–gel method. Ce^3+^ ions were then inserted into the pristine sample using Fe^3+^ ions with different concentrations: ZnCe_x_Fe_2−x_O_4_; X = 0.0–0.8; step = 0.02). We then investigated the antimicrobial and antibiofilm activities against some pathogenic microbes with the possible reaction mechanism determination (SEM imaging, and protein leakage assay) as well as anticancer effects of Eb and ZnCe_x_Fe_2−x_O_4_ nanoparticles (X = 0.0, 0.02, 0.04, 0.06, and 0.08) conjugated with Eb on human breast and colorectal cancer cell lines, evaluating their levels of ROS, inflammatory milieu, and related signalling effectors as well as investigating their effects on autophagy, cell cycle, and apoptosis.

## 2. Results and Discussion

### 2.1. Structural Studies

**Figure 1:**
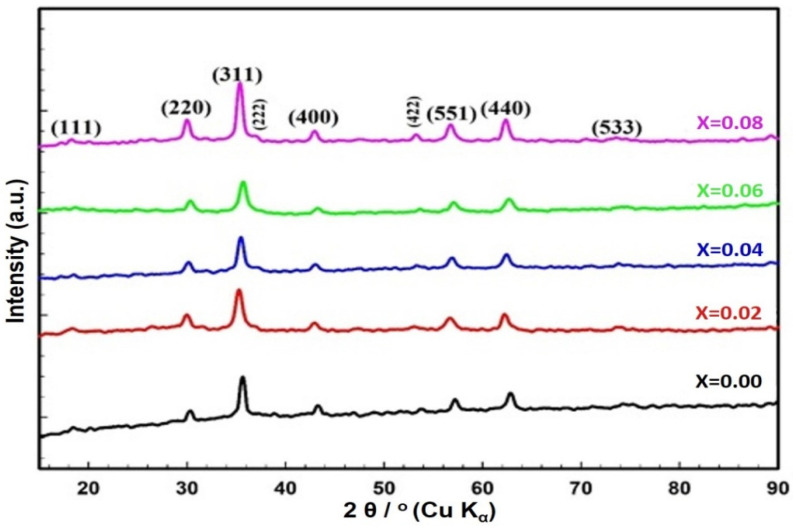
XRD patterns of ZnCe_x_Fe_2__−x_O_4_ (X = 0.0, 0.02, 0.04, 0.06, 0.08) NPs.

The X-ray diffraction (XRD) patterns of ZnCe_x_Fe_2−x_O_4_ (X = 0.0, 0.02, 0.04, 0.06, 0.08) NPs are presented in Figure 1. The diffraction peaks observed correspond to those of spinel ferrites, belonging to the Fd3 m space group (JCPDS card nos. 88-1935, and 74-2082) [23,24]. The crystallite size (D) ranged between 7.75 and 11.63 nm, and the lattice constant, a_exp_, was in the range 8.34–8.44 nm, as obtained in our previous work [25]. The change in a_exp_ and D is attributed to the replacement of the smaller ionic crystal radius of Fe^3+^ (0.064 nm) by the larger Ce^3+^ (0.103 nm) ions [26,27,28,29].

**Figure 2:**
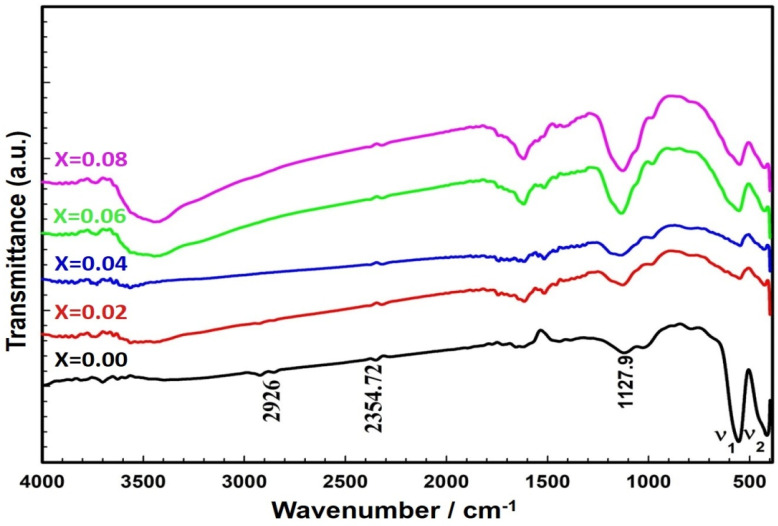
FTIR spectra of ZnCe_x_Fe_2−x_O_4_ (X = 0.0, 0.02, 0.04, 0.06, 0.08) samples.

Figure 2 shows the Fourier transform infrared (FTIR) spectra of ZnCe_x_Fe_2−x_O_4_ (X = 0.0, 0.02, 0.04, 0.06, 0.08) samples. For the ZFO spinel ferrite sample used in this work, there were two peaks, υ_1_ = 551.06 cm^−1^ and υ_2_ = 433.67 cm^−1^. These peaks are fundamental and confirm the successful formation of the cubic spinel phase in the ZFO sample [30,31,32]. The positions of the vibrational bands are listed in Table 1. In general, spinel ferrites show two essential vibrational bands, υ_1_ and υ_2_, which correspond to the stretching vibration of tetrahedral groups (A-site) and the stretching vibration of octahedral groups (B-site) [25]. From Figure 2, it is clear that the insertion of Ce^3+^ ions into the structure of ZnCe_x_Fe_2−x_O_4_ shifted the bands of the tetrahedral and octahedral sites toward the lower-frequency side. The substitution of Ce^3+^ ions into the B-site resulted in the migration of an equal number of Fe^3+^ and Zn ions from A sites to B sites to ease the strain [26]. The ionic radii of the B sites increased due to the settlement of the Ce^3+^ ions. This augmentation in the ionic radii of the A and B sites reduced the fundamental frequency [26,33]. The peaks with a wave number of 2354 cm^−1^ were attributed to the presence of carbonyl groups, while the peak at around 2926 cm^−1^ was ascribed to O-H stretching [10,11,12,25,34,35].

**Figure 3:**
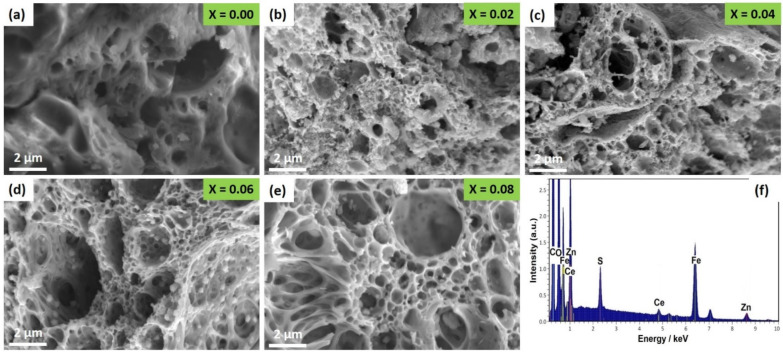
Surface morphology and elemental analysis where (**a**) SEM images of ZnCe_x_Fe_2−x_O_4_ NPs; X = 0.0, (**b**) X = 0.02, (**c**) X = 0.04, (**d**) X = 0.06, (**e**) X = 0.08), and (**f**) EDX analysis of ZnCe_x_Fe_2−x_O_4_ NPs.

The scanning electron microscopy (SEM) images of ZnCe_x_Fe_2−x_O_4_ (X = 0.0, 0.02, 0.04, 0.06, 0.08) NPs are shown in Figure 3a–e. The surface behaviour reveals an inhomogeneous grain appearance, in which the smooth agglomerates can be observed due to the occupation of a large quantity of Ce^3+^ ions at the grain boundary that could control the grain growth [36]. The surface was markedly porous and presented a coalescing form of the agglomerated particles connected with the interfacial surface tension phenomena [25]. The composition of the ZnCe_0.8_Fe_1.92_O_4_ sample was analysed using energy-dispersive X-ray spectra (EDX) (Figure 3f), and the presence of Ce, Zn, O, C, and Fe was confirmed [11,12]. 

**Figure 4:**
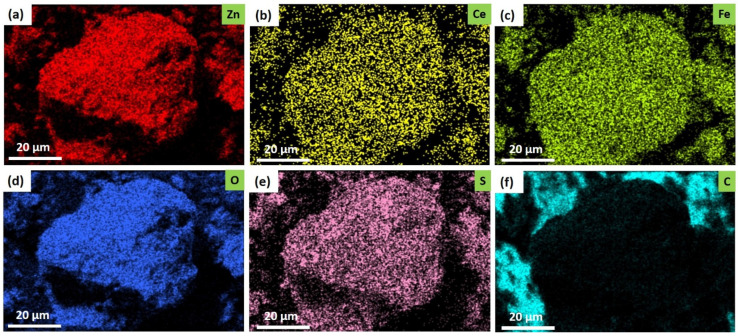
Elemental mapping images of ZnCe_x_Fe_2−x_O_4_ (X = 0.08) NPs, where (**a**) for Zn atom, (**b**) for Ce atom, (**c**) for Fe atom, (**d**) for O atom, (**e**) for S atom, and (**f**) for C atom.

To further illustrate the structural features of the samples, mapping of elements to the ZnCe_0.8_Fe_1.92_O_4_ sample was carried out (Figure 4). It is evident from these images that the elements Zn, Fe, Ce, C, S, and O exist, an observation that agreed with the preceding EDX results. These elements were homogeneously distributed.

**Figure 5:**
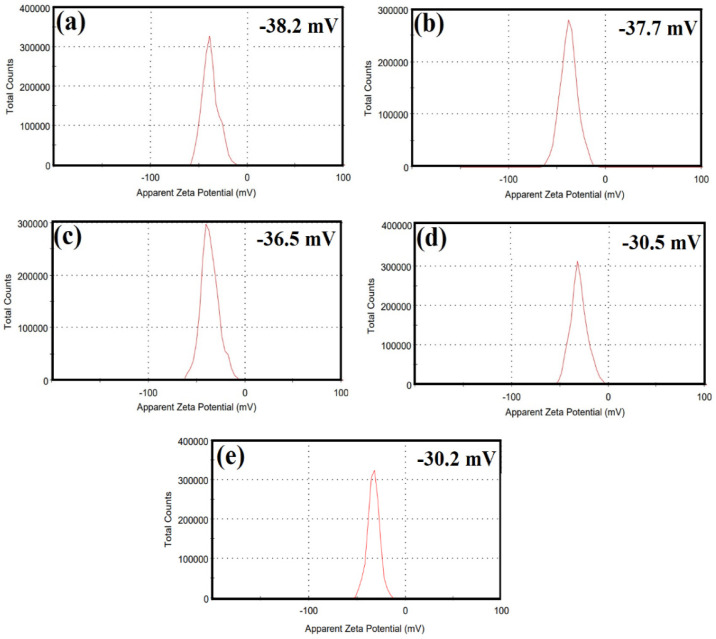
Surface charge determination of ZnCe_x_Fe_2−x_O_4_ by Zeta potential when (**a**) X = 0, (**b**) X = 0.02, (**c**) X = 0.04, (**d**), X = 0.06, and (**e**) X = 0.08 at pH 7 (cultural media pH).

The Zeta potential of the synthesised ZnCe_x_Fe_2−x_O_4_ (X = 0.0, 0.02, 0.04, 0.06, 0.08) NPs was examined at the culture media as used in the treatments to determine the surface charge of the synthesised samples, which in turn determine the stability, as observed in Figure 5. From the present results, it is notable that the Zeta potential of the ZnCe_x_Fe_2−x_O_4_ (X = 0.0, 0.02, 0.04, 0.06, 0.08) NPs surface maintains a negative statement at pH 7 (cultural media pH). The initial Zeta potential of ZnFe_2_O_4_ NPs was −38.2 mV (Figure 5a), which completely agrees with the previous results obtained [37,38]. After the substitution of Ce^3+^ on ZnFe_2_O_4_ NPs (at different concentrations), the potential of substituent samples was slightly changed to be −37.7, −36.5, −30.5, and −30.2 mV when X = 0.02, 0.04, 0.06, and 0.08, respectively, due to the positive charge of Ce^3+^ and the net charge still negative at neutral medium (pH 7), as shown in Figure 5b,c,d,e, respectively. The magnitude of the zeta potential is predictive of the colloidal stability [39]. Nanoparticles with Zeta potential values from ±30 to ±40 have moderate stability [40], as shown in our synthesised samples. Dispersions with a low zeta potential value will eventually aggregate due to the Van Der Waal inter-particle attractions [41].

**Figure 6:**
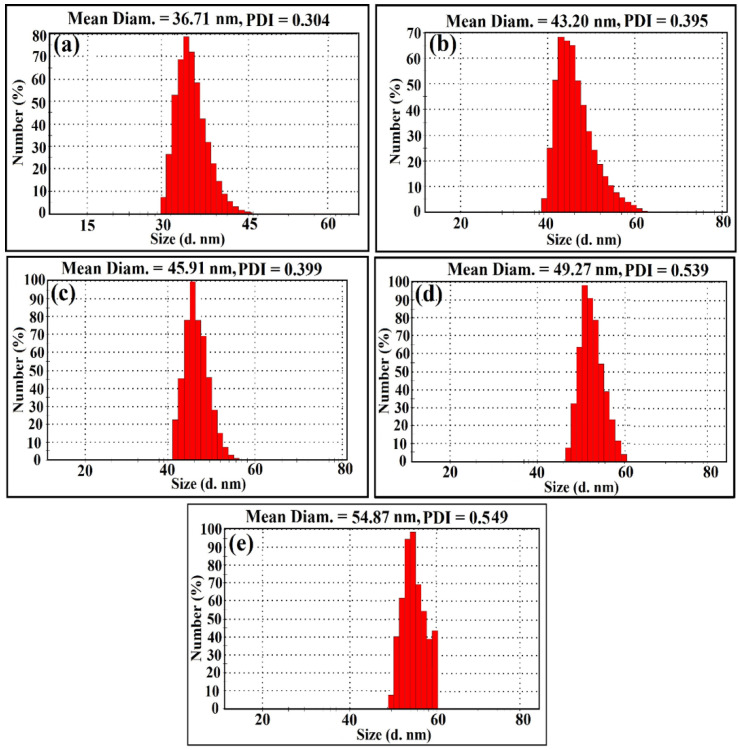
Particle size distribution and PDI determination of ZnCe_x_Fe_2−x_O_4_ by DLS when (**a**) X = 0, (**b**) X = 0.02, (**c**) X = 0.04, (**d**) X = 0.06, and (**e**) X = 0.08 at pH 7 (cultural media pH).

Dynamic light scattering (DLS) analysis was performed to evaluate particle size distribution and to calculate the average particle size of the synthesised ZnCe_x_Fe_2−x_O_4_ (X = 0.0, 0.02, 0.04, 0.06, 0.08) NPs that was found as 36.71 nm when X = 0. Additionally, the particle size was found to be 43.20, 45.91, 49.27, and 54.87 nm when X = 0.02, 0.04, 0.06, and 0.08, respectively, in the synthesised ZnCe_x_Fe_2−x_O_4_ NPs, as shown in Figure 6.

It is important to state that the grown moderate mono-size distributed ZnCe_x_Fe_2−x_O_4_ (X = 0.0, 0.02, 0.04, 0.06, 0.08) NPs were attributed to the synthesis method, and the particle size distribution was sharply increased as Ce^3+^ ions content increased in the prepared sample. It is common that DLS size measurements become higher than the crystallite size (D) measurements (Figure 1), as DLS analysis is estimating the hydrodynamic radius of ZnCe_x_Fe_2−x_O_4_ (X = 0.0, 0.02, 0.04, 0.06, 0.08) NPs bounded by water molecules, resulting in larger particle sizes of the capped NPs, while XRD analysis is calculating the crystallite size of the powder material without water layer [42].

The polydispersity index (PDI) can be obtained from instruments that use DLS or are determined from electron micrographs. International standards organisations (ISOs) have established that PDI values < 0.05 are more common to monodisperse samples, while values > 0.7 are common to a broad size (e.g., polydisperse) distribution of particles [43]. Herein, for the obtained PDI values (Figure 6), we found that the PDI value increases as Ce^3+^ ions content increases in the prepared sample and was found to be 0.304, 0.395, 0.399, and 0.539 in ZnCe_x_Fe_2−x_O_4_ NPs when X = 0.0, 0.02, 0.04, 0.06, 0.08, respectively. The present values indicate that the synthesised samples were moderate mono-size distributed. 

### 2.2. Antimicrobial Potential

Antimicrobial agents have been used to treat and control the microbial infection [44]. The use of novel nanomaterial-based agents for the limitation of pathogenic microbes has received attention from several researchers [10]. In our study, the as-synthesised ferrite samples were checked for their antimicrobial activity using the agar-disc diffusion technique. The results indicated that the ferrite specimens reduced the activity of a wide range of bacteria including *P. aeruginosa*, *P. mirabilis*, and *S. aureus*. ZnCe_x_Fe_2−x_O_4_; X = 0.06 had the most powerful antimicrobial effects against all the microbes examined. The power of ZnCe_x_Fe_2−x_O_4_; X = 0.06 as antimicrobial agent declines in the following order: *C*. *albicans* (13.5 ± 0.5000 mm), ˃*S*. *aureus* (13.2 ± 0.2335 mm), ˃*P.* *aeruginosa* (9.5 ± 0.5000 mm), ˃*P. mirabilis* (9.5 ± 1.0000 mm), ˃*P. vulgaris* (9.0 ± 0.2335 mm), ˃*S. typhi* (9.0 ± 0.6545 mm), ˃*C. tropicalis* (8.9 ± 0.6545 mm), ˃*E. coli* (8.5 ± 0.6545 mm), and ˃*K. pneumoniae* (8.0 ± 0.5755 mm) (Table 2).

**Figure 7:**
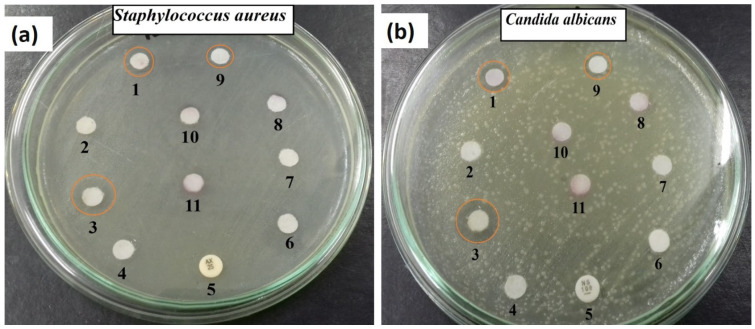
Antimicrobial activities and ZOI (mm) of tested ferrites (ZnCe_x_Fe_2−x_O_4_) against the pathogenic microbes *Staphylococcus aureus* (**a**) and *Candida albicans* (**b**), where (1) X = 0.08, (2) X = 0.02, (3) X = 0.06, (4) X = 0.00, (5) standard positive control as amoxicillin (AX), and nystatin (NS), (6) DMSO (negative control), (7) Fe(NO_2_)_3_·9H_2_O, (8) ZnSO_4_.7H_2_O, (9) X = 0.04, (10) Ce(NO_3_)_3_.6H_2_O, and (11) Eb; circle indicating the presence of ZOI.

The antimicrobial activity of the synthesised ZnCe_x_Fe_2−x_O_4_ increased as the X value increased (Figure 7a). The highest zone of inhibition (ZOI) of *S. aureus* was observed when X = 0.06 in ZnCe_x_Fe_2−x_O_4_ and slightly decreased when X = 0.08. In the case of *C. albicans*, the active sample was ZnCe_x_Fe_2−x_O_4_ with X =0.06 (Figure 7b).

The antimicrobial abilities of the as-synthesised samples were compared with the standard antibacterial amoxicillin (AX; 25.0 μg/mL) and the antifungal agent nystatin (NS; 25.0 μg/mL) as a positive control. Our samples were more active than the standard antibiotics, and the microbes tested were resistant to the standard antibiotics. The synthesized ferrites samples (5.0 µL; 1.0 µg/mL) were placed over 6.0 mm applied disks.

Next, nanoparticles were compared with the precursors used in the synthetic process, Eb alone and the dimethylsulfoxide (DMSO) organic solvent (as a negative control), and not all were active against the microbes tested (Figure 7a,b).

The synthesised ZnCe_x_Fe_2−x_O_4_; X = 0.06 was more active against Gram-positive bacteria than against Gram-negative bacteria because the cell wall of Gram-negative bacteria contains a thick layer of lipopolysaccharides in addition to a small layer of peptidoglycans, whereas Gram-positive bacteria have a thicker layer of peptidoglycans [45]. In general, inorganic NPs have high surface-to-volume ratios and nanoscale sizes. Consequently, they can combine and interact with some pathogenic microbes such as yeasts, bacteria, and fungi [46]. The unique properties of the inorganic NPs make them potentially valuable in a wide range of biomedical applications. With the decreases in the effectiveness of traditional antibiotics due to the increases in drug resistance in some bacteria, NPs may be valuable as medications [47].

The results of the minimum inhibitory concentrations (MIC) tests (ranged from 0.195 to 12.50 μg/mL) of the samples against all microbes were tested. The MIC of ZnCe_x_Fe_2−x_O_4_; X = 0.06 was 0.390 μg/mL against *S. aureus*. The synthesised ZnCe_x_Fe_2−x_O_4_; X = 0.06 had an MIC of 0.195 μg/mL against *C. albicans*, suggesting that it could be used as an antifungal agent at low concentration, which means that the minimum concentration of our synthesised sample gave antimicrobial activity that was less than 0.5 part per million (ppm), which is a good and promising result, especially as they will not have any toxicity when applied in the in vivo studies.

The properties of the synthesised ferrites play a vital role in their antimicrobial characteristics: their elemental structure, purity, and size of the synthesised ferrites must be analysed to explain their antimicrobial activity [48]. The composition of the ferrites, their particle size, and the doping with Ce played an important part in improving the antimicrobial efficacy of the ZnCe_x_Fe_2−x_O_4_ at very low concentrations (10.0 µg/mL) against all tested bacteria and yeasts. They possess encouraging physical and chemical behaviour, more than those of the usual organic and synthetic antimicrobial agents, such as a unique link to pathogens, leading to more interaction with pathogenic bacteria and yeasts and therefore increasing their antimicrobial potential [49]. The mechanisms of action were enhanced by the ability of the NPs to modify the distribution of ROS such as the superoxide anion O_2_^−^ [50], the infiltration of ZnCe_x_Fe_2−x_O_4_ within the pathogenic microbes, and an alkaline tendency [51]. ZnCe_x_Fe_2−x_O_4_ might be able to alter the microbial morphology and the composition of the biofilms, change the microbial membrane permeability, and induce expression of the oxidative stress genes [52]. 

### 2.3. Antibiofilm Activity of ZnCe_x_Fe_2−x_O_4_; X = 0.06

The production of biofilms by pathogenic microbes is characterised by the secretion of exo-polysaccharides [53]. The test tube method was applied to determine the antibiofilm potential of the synthesised ferrites against some familiar pathogenic microbes.

**Figure 8:**
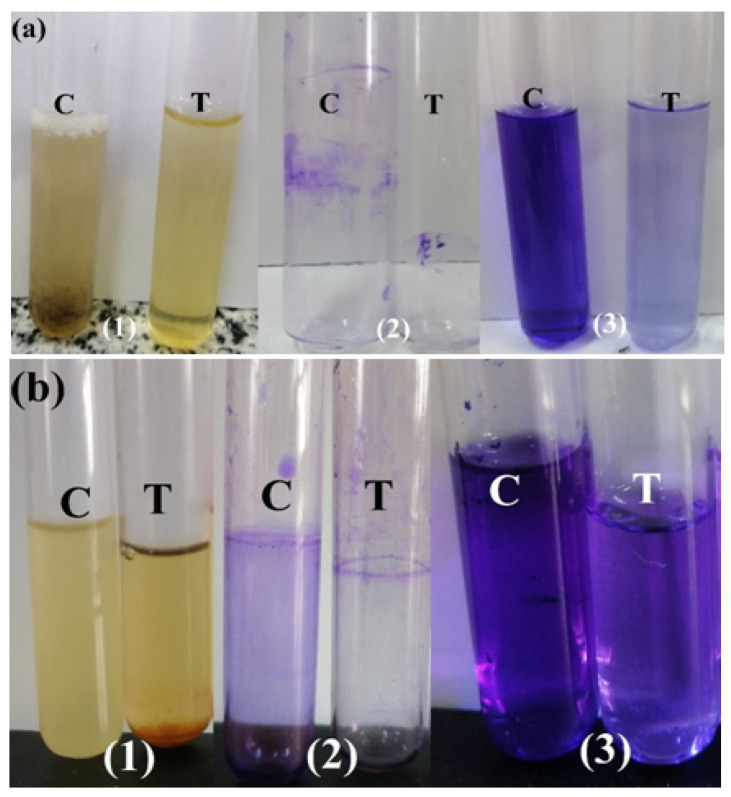
Antibiofilm potential of the synthesised ZnCe_x_Fe_2−x_O_4_; X = 0.06 using the tube method against *Staphylococcus aureus (***a**) and *Candida albicans* (**b**); where the steps are reported as follows: (1) growth of the bacterial and yeast cells and biofilm formation (rings) without the treatment with the synthesised ZnCe_x_Fe_2−x_O_4_; X = 0.06 and inhibition of the bacterial and yeast growth after treatment with ZnCe_x_Fe_2−x_O_4_; X = 0.06; (2) staining of the adherent bacterial and yeast cells with crystal violet, and (3) removal and dissolution of the adherent bacterial and yeast cells by ethanol for semi-quantitative biofilm inhibition determination.

Figure 8 shows the antibiofilm action of the as-synthesised ZnCe_x_Fe_2−x_O_4_; X = 0.06 against *S. aureus* and *C. albicans.* The complete steps were (I): normal microbial growth and production of a distinct ring in the absence of the synthesised ZnCe_x_Fe_2−x_O_4_; X = 0.06 and interference with microbial growth in the vicinity of ZnCe_x_Fe_2−x_O_4_; X = 0.06, (II): staining of the biofilm with crystal violet (CV), which produced qualitative results, and (III): elimination and separation of the adhered microbial cells after the addition of ethanol for the semi-quantitative measurement of the extent of biofilm hindrance (Table 3).

Figure 8a displays the start of the tube design for the determination of the antibiofilm potential of ZnCe_x_Fe_2−x_O_4_; X = 0.06 against *S. aureus.* This bacterium produced a thick whitish-yellow layer at the air–liquid interface in the ZnCe_x_Fe_2−x_O_4_; X = 0.06 control. The matte layers produced were fully adhered across the walls of the tubes and developed a blue colour following staining with CV. A dark blue colour was created in the solution subsequent to dissolving CV with absolute ethanol (Figure 8a).

The managed tubes that included *S. aureus* cells and 10.0 µg/mL ZnCe_x_Fe_2−x_O_4_; X = 0.06 showed a marked negative effect on biofilm and ring formation. The colour of the adherent cells was muted, and the blue colour was faint after the addition of ethanol (Figure 8a). Similar results were observed for the repression of biofilms of the yeast *C. albicans* (Figure 8b).

The semi-quantitative determination of the inhibition percentage was performed using a UV-Vis spectrophotometer at 570.0 nm. The optical density (O.D) was estimated following the elimination of CV-stained biofilms.

Table 3 discerns the percentage of inhibition. The highest percentage was observed for *S. aureus* (92.73%), *P. mirabilis* (79.54%), and *C. albicans* (90.18%) following the addition of 10.0 µg/mL ZnCe_x_Fe_2−x_O_4_; X = 0.06.

ZnCe_x_Fe_2−x_O_4_; X = 0.06 controlled the growth of biofilm at a constant degree of adhesion, the first step in the antimicrobial behaviour [54]. The difference in the inhibitory percentage may be produced by many factors, such as the potential of the antimicrobial agents, the attraction on the surface because of the large surface area of the ZnCe_x_Fe_2−x_O_4_; X = 0.06, physical features such asZnCe_x_Fe_2−x_O_4_ particle sizes, invasion skills, and different chemical characteristics influencing the relationship and communication of ZnCe_x_Fe_2−x_O_4_ with biofilm-producing microbes. ZnCe_x_Fe_2−x_O_4_; X = 0.06 repressed the growth of *S. aureus* by more than 98% at 0.390 µg/mL, as mentioned in the MIC results. By arresting exo-polysaccharide synthesis, which is a precursor to biofilm formation, the creation of *S. aureus* biofilm was then prevented [53].

### 2.4. Reaction Mechanism Determination Using SEM Analysis

**Figure 9:**
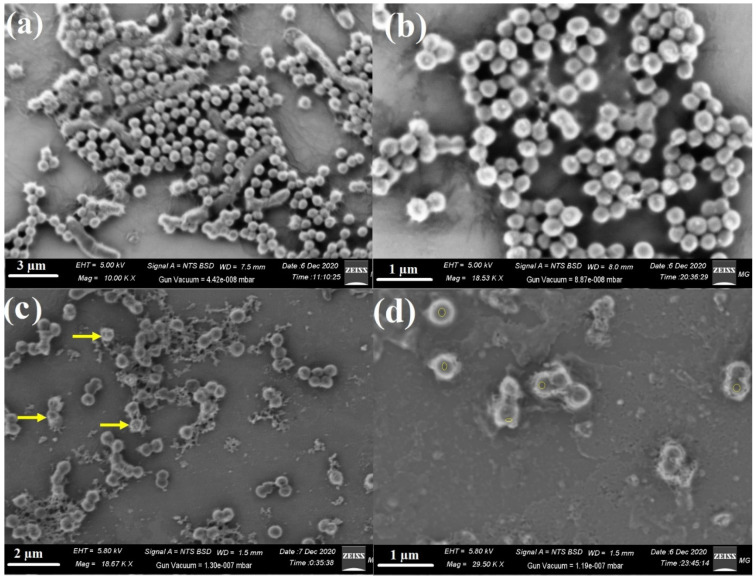
SEM images of *S. Aureus*; (**a**) regular bacterial cells (*S. aureus*) without ZnCe_x_Fe_2−x_O_4_; X = 0.06 treatment, (**b**) other magnified area regarding control *S. aureus*, (**c**) malformed and an irregular bacterial cells after ZnCe_x_Fe_2−x_O_4_; X = 0.06 treatment showing full lysis (yellow arrows), and (**d**) other magnified area regarding treated *S. aureus* showing the formation of pits on the bacterial surface (yellow circles).

To explain the antimicrobial behaviour of the synthesised ZnCe_x_Fe_2−x_O_4_; X = 0.06, we tried to define the mechanism of action toward *S.* *aureus* after the SEM analysis. The SEM analysis showed the appearance of the bacterial cells (*S.* *aureus*) following ZnCe_x_Fe_2−x_O_4_; X = 0.06 treatment of the control sample. In the control sample, bacterial groups were constantly developed and displayed typical cellular forms, including the normal bacterial surface and semi-formed biofilm (Figure 9a).

After treatment with ZnCe_x_Fe_2−x_O_4_; X = 0.06, morphological differences in *S.* *aureus* were apparent (Figure 9b). We also observed the complete lysis of bacterial cells with a concomitant decrease in the viable number, and ultimately the growth of biofilm was inhibited (Figure 9b). These results reflected the antimicrobial activity of Ce addition in the synthesised ZnCe_x_Fe_2−x_O_4_; X = 0.06 and confirmed the ZOI results (Table 2).

### 2.5. Determination of Protein Leakage from Bacterial Cell Membranes

**Figure 10:**
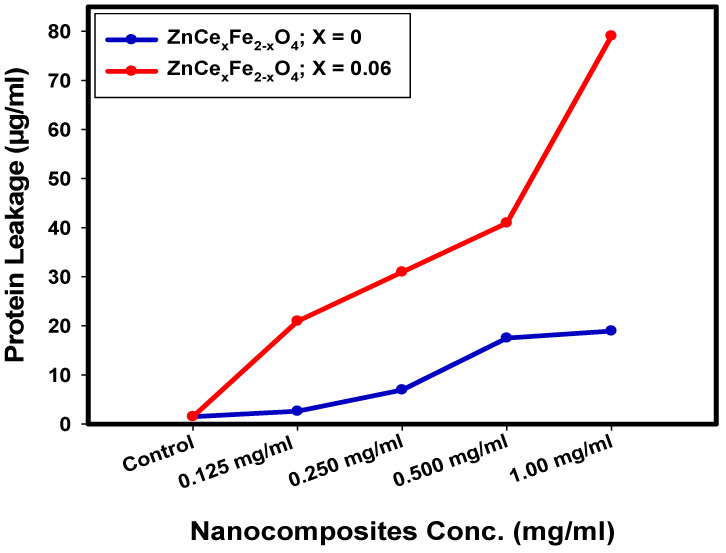
The effect of ZnCe_x_Fe_2−x_O_4_; X = 0 and 0.06 on the protein leakage from *S. aureus* cell membranes.

The quantities of protein discharged in the suspension of the treated *S. aureus* cells were determined applying the Bradford method [55]. As shown in Figure 10, it is obvious that the quantity of cellular protein discharged from *S. aureus* is directly proportional to the concentration of ZnCe_x_Fe_2−x_O_4_; X = 0.06 nanocomposite and was found to be 79.05 µg/mL after the treatment with 1.0 mg/mL of the tested ZnCe_x_Fe_2−x_O_4_; X = 0.06 nanocomposite, which proves the antibacterial characteristics of the synthesised nanocomposites and explains the creation of holes and destruction in the cell membrane of *S. aureus*, causing the oozing out of the proteins from the *S. aureus* cytoplasm. On the other hand, the synthesised ZnCe_x_Fe_2−x_O_4_; X = 0 nanocomposite exhibited reduced activity in membrane leakage after measured cellular protein release from *S. aureus* and was found to be 18.95 µg/mL. 

These results revealed that ZnCe_x_Fe_2−x_O_4_ nanocomposite; where X = 0.06 showed an improvement in the permeability of *S. aureus* cell membranes more than ZnCe_x_Fe_2−x_O_4_; where X = 0 nanocomposite. Therefore, it could be assumed that confusion of membranous permeability would be a vital portion of the repression of bacterial mass. Related studies [56] and [57] described comparable outcomes when ferrites were incorporated, which revealed concentration-dependent destabilisation in the cell membrane of bacterial cells and pointed to leakage of their intracellular substance into the extracellular form (bacterial cell suspension). 

Paul et al. [58] proved that the difference in bacterial cell membrane permeability was shown in percentage difference in corresponding electric conductivity. It was reported that the percentage of relative electric conductivities of all tested samples improves with the rise in the concentration of the treated nanocomposites. The integrity of the bacterial cell membrane is defined by analysing the discharge of bacterial cell components such as proteins; the leakage developed with time, as there was constant cell membrane injury that pointed to the leakage of cell components deriving from the cell destruction, which confirms the results obtained in SEM analysis (Figure 9).

**Figure 11:**
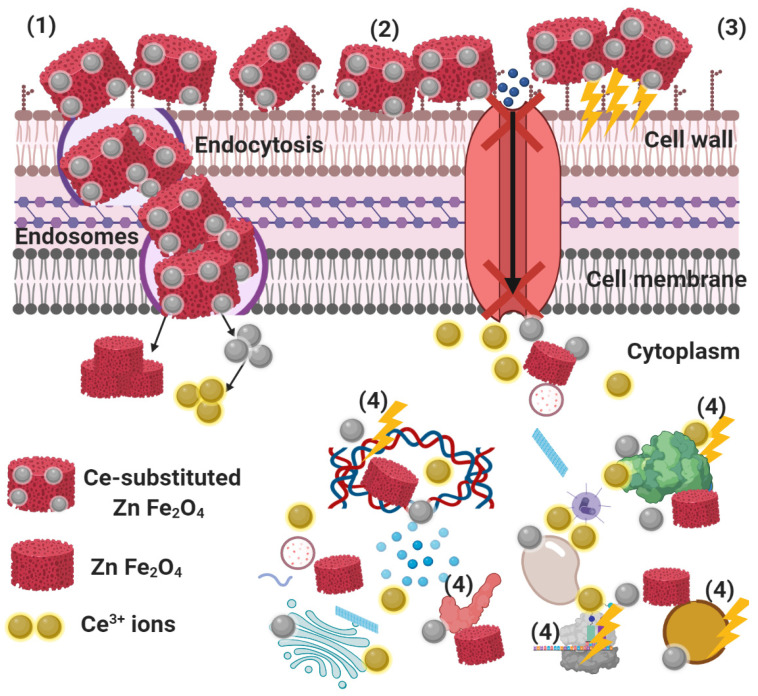
Proposed schematic representation illustrating the four prominent methods of antimicrobial activity of ZnCe_x_Fe_2−x_O_4_; X = 0.06, where (**1**) ZnCe_x_Fe_2−x_O_4_; X = 0.06 adheres to the bacterial cell surface and results in membrane damage and altered transport activity; (**2**) ZnCe_x_Fe_2−x_O_4_; X = 0.06 creates and increases ROS, leading to cell damage; (**3**) ZnCe_x_Fe_2−x_O_4_; X = 0.06 blocks the transport of ions to and from the bacterial cell; and (**4**) ZnCe_x_Fe_2−x_O_4_; X = 0.06 penetrates the bacterial cells and interacts with cellular organelles and biomolecules, affecting the cellular machinery, modulating the cellular signal system, and causing cell death. ZnCe_x_Fe_2−x_O_4_; X = 0.06 may serve as a vehicle to effectively deliver Ce^3+^ ions to the bacterial cytoplasm and membrane, where a proton motive force would decrease the pH to less than 3.0 and therefore improve the release of Ce^3+^.

El-Batal et al. [59] have shown that there are four mechanisms that produce the effects of metal NPs on microbial cells. After comparison with our study, we recognise that ZnCe_x_Fe_2−x_O_4_; X = 0.06 start their activity by adhesion at the outer surface of the microbial cell, allowing membrane damage, formation of pits (as mentioned in membrane leakage assay), and switching off of the ions’ transport activity (Figure 11).

The nano-metals then modify the ionic structure (Ce^3+^) inside the bacterial cell at pH 3 and interfere with the intracellular structures such as plasmids, DNA, and other vital organelles. Cellular toxicity occurs due to the oxidative stress generated by the production of ROS (Figure 11). ZnFe_2_O_4_ NPs could withstand the acidic conditions inside the bacterial cells, and the conversion described above did not occur [60], but the antibacterial effect was caused by the presence of nano-structures inside the bacterial cells, which, in turn, affected signal transduction pathways. There were significant reaction mechanisms such as reactive oxygen species (ROS) division (superoxide anion; O_2_^−^) [59], and it is suggested that ZnCe_x_Fe_2−x_O_4_; X = 0.06, could alter the microbial morphology, diminish the microbial membrane permeability, and induce the abundance of oxidative stress genes as a compensatory response due to the H_2_O_2_ production [49,59].

### 2.6. Antitumour Activity of Eb-ZFO and Eb-ZnCe_x_Fe_2−x_O_4_; X = 0.06 Nanocomposites with or without γ-Radiation

#### 2.6.1. Screening of the Cytotoxic Profile of Different Concentrations of Eb and ZnCe_x_Fe_2−x_O_4_ Nanocomposites on Human Breast Cancer (MDA-MB-231; Triple-Negative Basal B Subtype) and Colon Cancer (HT-29; Colorectal Adenocarcinoma)

**Figure 12:**
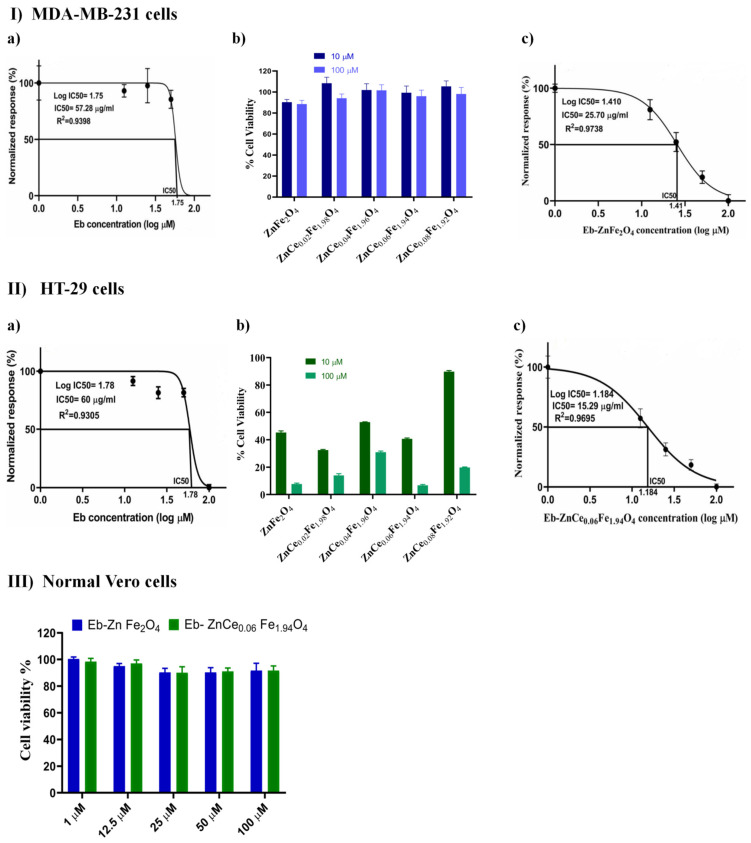
Cytotoxicity screening of various concentrations of Ebselen (Eb) and/or ZnCexFe_2_-XO_4_ nanoparticles. (**I**) MDA-MB-231 cells treated as follows: (**a**) Eb, (**b**) ZnCexFe_2_-XO_4_, and (**c**) Eb-ZnFe_2_O_4_. (**II**) HT-29 cells treated as follows: (**a**) Eb, (**b**) ZnCexFe_2_-XO_4_, and (**c**) Eb-ZnCe_0.06_Fe_1.94_O_4_. (**III**) Normal Vero cells treated by Eb-ZnFe_2_O_4_ and Eb-ZnCe_0.06_Fe_1.94_O_4_ at a concentration of 100 µM. The percent survival was calculated based on untreated cells of both cell lines and was set at 100% (n = 3). Inverted light microscopy images of all the above tests are represented as shown in Figure S1 (I, II and III).

The cytotoxicity screening found the half-maximal inhibitory concentration (IC_50_) of Eb to be 57.28 µg/mL on MDA-MB-231 cells (Figure 12I(a)) and 60 µg/mL on HT-29 cells (Figure 12II(a)). The optimal cytotoxic concentration of ZnCe_x_Fe_2−x_O_4_ nanoparticles at different concentrations of (X) on MDA-MB-231 and HT-29 cell lines were 100 µM/L for ZnFe_2_O_4_ (Figure 12I(b)) and 100 µM/L for ZnCe_0.06_ Fe_1.94_ O_4_ (Figure 12II(b)), respectively. Magnetic nanoparticles have valuable properties as theranostics, including hyperthermia and magnetic resonance imaging (MRI), and can be used in biosensors and drug delivery platforms [61,62]. Saquib et al. [63] reported that ZnFe_2_O_4_ NPs possess antitumour potential via the induction of apoptosis and necrosis in human amnion epithelial (WISH) cells, through the mitochondria-dependent intrinsic apoptotic pathway. It has also been found that ZnFe_2_O_4_ NPs cause genomic instability in the meristematic root cells of sunflowers, through induced chromosomal aberrations [64], supporting the contention that ZnFe_2_O_4_ NPs are cytotoxic. Several studies have found Ce oxide to have a unique electronic configuration, providing anti-inflammatory, non-invasive, and oxidative stress features. These characteristics result in the production of ROS at the microvascular stage level, owing to their natural reduction and oxidation reactions in the cells [65,66]. ROS generation relies on the production of defects caused by oxygen vacancies in the crystal structure of the nanoparticles, which could be boosted by selective metal ion doping of the lattice structure [67]. Apart from the stabilised dissolution of Zn from the ZnFe_2_O_4_ lattice owing to Fe doping, which contributes to the tumour cell growth inhibition, CeO_2_ doping with metals resulted in an increased photocatalytic activity, due to a better separation of h^+^/e^-^ pairs [68] owing to the electron accepting capability and/or hole donors, and facilitates charge carrier localisation [69]. In turn, these migrated holes contribute to the production of ·OH radicals when reacted with chemisorbed H_2_O molecules and form the free radicals ·OH and O_2_^.^, which are the primary cause of cell death and the oxidation of organic matter such as bacterial cell walls and membranes [70]. This phenomenon might explain the potentiated cytotoxic activity of ZnFe_2_O_4_ lattice after CeO_2_ doping observed in the current data. Furthermore, the antitumour efficacy of Eb, an organo-selenium compound, is attributed to its ability to induce apoptosis, inhibit angiogenesis, upregulate caspases and DNA fragmentation, cause cell-cycle arrest, and reduce oxidative stress in many cancers [71,72]. The IC_50_ of Eb was taken into account when determining the optimal concentration of ZnFe_2_O_4_ nanocarrier, which was then incorporated into the optimal concentration of ZnFe_2_O_4_, afterwards applied to MDA-MB-231 cells, and the IC_50_ was found to be 25.7 µg/mL (Figure 12I(c)). In HT-29 cells, the previously determined IC_50_ of Eb was incorporated into the optimal cytotoxic concentration in the ZnCe_0.06_Fe_1.94_O_4_ nanocarrier, and the IC_50_ was found to be 15.29 µg/mL (Figure 12II(c)). The incorporation of Eb to ZnCexFe_2_-xO_4_ nanocomposite enhanced the cytotoxic effect against cancers cell lines, as shown in our study. However, incubation of normal Vero renal epithelial cells with Eb-ZnFe_2_O_4_ and Eb-ZnCe_0.06_Fe_1.94_O_4_ at concentrations ranging from 1–100 µM over 24 h showed no cytotoxicity or morphological changes (Figure 12III) versus MDA-MB-231 and HT-29 cells, which revealed a notable greater susceptibility against both NPs at corresponding concentrations. The morphological alterations represented reduced cell viability and population, detachment, rounding, and shrinkage, suggesting the incidence of apoptosis (as shown in Figure S1-I, II, and III). Accordingly, these data suggest the selective toxicity against MDA-MB-231 and HT-29 cells.

#### 2.6.2. ROS Status and Signaling Molecules ERK1/2, JNK and NRF-2 in MDA-MB-231 and HT-29 Cells

##### MDA-MB-231 Cell Line

**Figure 13:**
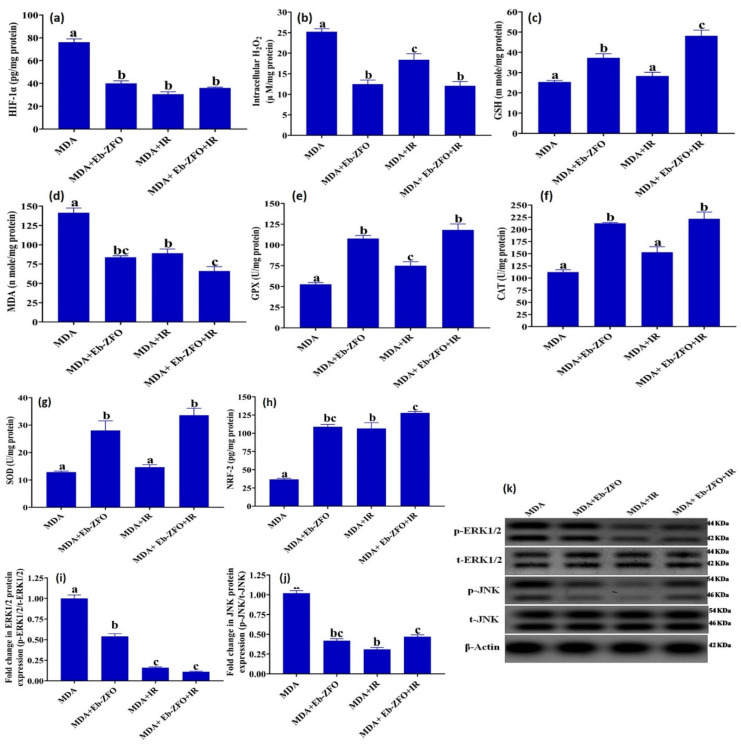
ROS status and associated signalling molecules ERK1/2, JNK, and NRF-2 in the MDA-MB-231 cell line. ROS status indicators were the levels of (**a**) HIF-1α, (**b**) intracellular H_2_O_2_, (**c**) GSH, and (**d**) MDA; activities of (**e**) GPX, (**f**) CAT, and (**g**) SOD. ROS-sensing signalling molecules were (**h**) NRF-2 level; fold change in protein expression of (**i**) ERK1/2, (**j**) JNK, and (**k**) representative western blot analysis, SDS-PAGE of ERK1/2, JNK, and β- actin. Results are expressed as the mean and standard error of the mean (SEM) (n = 3). Columns with dissimilar letters (a, b, c…) overhead within the same histogram are significantly different, and columns that have the same letters are not significantly different at *p* < 0.05. MDA group: untreated MDA-MB-231 cells served as control, MDA + Eb-ZFO group: MDA-MB-231 cells treated with Ebselen (Eb) and ZnFe_2_O_4_ nanoparticles (ZFO), MDA + IR group: MDA-MB-231 cells exposed to γ-radiation; and MDA + Eb-ZFO + IR group: MDA-MB-231 cells treated with Eb-ZFO and exposed to IR.

The effect of Eb-ZFO on ROS status including hypoxia-inducible factor-1 alpha (HIF-1α), intracellular hydrogen peroxide (H_2_O_2_), malondialdehyde (MDA), and glutathione(GSH) levels as well as superoxide dismutase (SOD), catalase (CAT), and glutathione peroxidase (GPX) activities, and the associated signalling molecules extracellular signal-regulated kinases 1 and 2 (ERK1/2), Jun N-terminal Kinase (JNK), and nuclear factor erythroid 2-related factor 2 (NRF-2) in MDA-MB-231 cells are shown in Figure 13. The data for the MDA + Eb-ZFO group showed a significant reduction (*p* < 0.05) in the levels of HIF-1α (47.41%), intracellular H_2_O_2_ (50.52%), MDA (40.81%), the protein expression of p-ERK1/2 (46.03%), and p-JNK (58.82%) associated with a significant elevation in antioxidant system NRF-2 (2.96-fold) and GSH levels (1.47-fold) as well as SOD (2.17-fold), CAT (1.89-fold), and GPX (2.04-fold) activities when compared to the MDA group (MDA-MB-231 untreated cancer cells).

The exposure of MDA-MB-231 cells to γ-radiation (IR) produced insignificant changes in SOD and CAT activities. GSH levels showed a significant decrease (*p* < 0.05), as did the levels of HIF-1α (59.84%), intracellular H_2_O_2_ (26.98%), MDA (37.03%), the protein expression of p-ERK1/2 (83.99%), and p-JNK (69.54%), paralleled by a significant increase in NRF-2 level (1.89-fold) and GPX (0.43-fold) activity when compared to the MDA group (Figure 13).

The data from the MDA + Eb-ZFO + IR group revealed a significant decline (*p* < 0.05) in the levels of HIF-1α (52.69%), intracellular H_2_O_2_ (52.10%), MDA (53.31%), and the protein expression of p-ERK1/2 (87%) and p-JNK (65%) associated with a significant upregulation in antioxidant system in terms of NRF-2 (3.48-fold) and GSH levels (1.89-fold) along with SOD (2.60-fold), CAT (1.97-fold), and GPX (2.24-fold) activities when compared to the MDA group. The combination of Eb-ZFO with IR induced a significant modulation (*p* < 0.05) in ROS/antioxidant machinery imbalance, and the fine-tuning of redox status, compared to either each one alone and the MDA group (Figure 13).

##### HT-29 Cell Line

**Figure 14:**
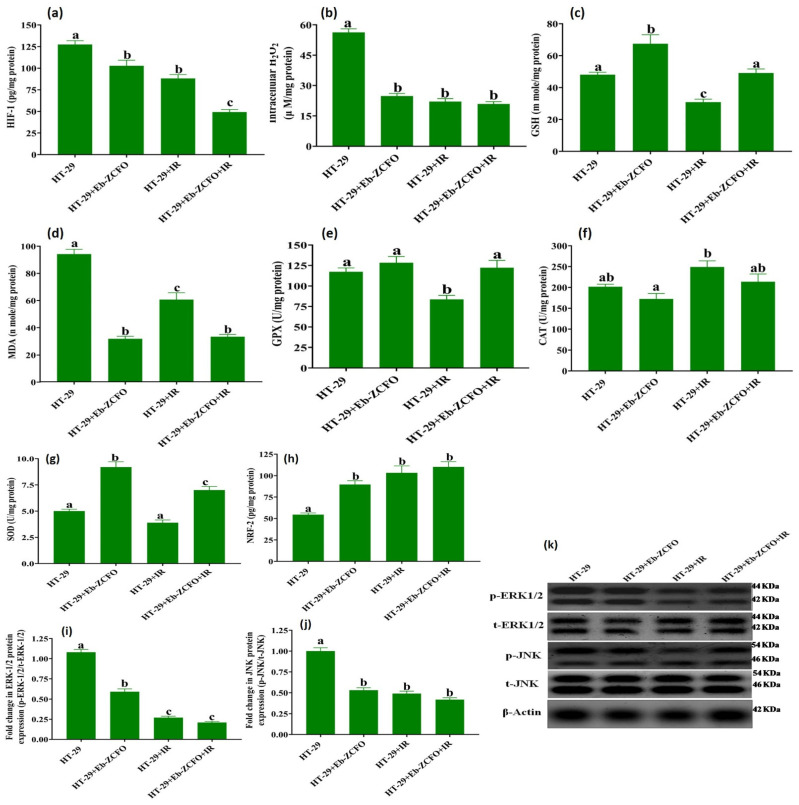
ROS status and associated signalling molecules ERK1/2, JNK, and NRF-2 in HT-29 cells lines. ROS status measured as follows: the levels of (**a**) HIF-1α, (**b**) intracellular H_2_O_2_, (**c**) GSH, and (**d**) MDA; activities of (**e**) GPX, (**f**) CAT, and (**g**) SOD. ROS-sensing signalling molecules as follows: (**h**) NRF-2 level; fold change in protein expression of (**i**) ERK1/2, (**j**) JNK, and (**k**) representative western blot analysis, SDS-PAGE of ERK1/2, JNK, and β-actin. Results are expressed as the mean and standard error of the mean (SEM) (n = 3). Columns with dissimilar letters (a, b, c…) overhead within the same histogram are significantly different, and columns with the same letters are not significantly different at *p* < 0.05. HT-29 group: untreated HT-29 cells served as control, HT-29 + Eb-ZCFO group: HT-29 cells treated with Ebselen (Eb) and ZnCe_0.06_Fe_1.94_O_4_ nanoparticles (ZCFO), HT-29 + IR group: HT-29 cells exposed to γ-radiation, and HT-29 + Eb-ZCFO + IR group: HT-29 cells treated with Eb-ZCFO and exposed to IR.

As shown in Figure 14, the effect of Eb-ZCFO on ROS status and the levels of the signalling molecules ERK1/2, JNK, and NRF-2 in the HT-29 cell line showed a significant reduction (*p* < 0.05) in the levels of HIF-1α (19.39%), intracellular H_2_O_2_ (55.95%), and MDA (66.24%), as well as p-ERK1/2 (45.39%) and p-JNK (47.00%) protein expression associated with a significant elevation in the antioxidant system (NRF-2 1.64-fold, SOD 1.84-fold, and GSH 1.40-fold) along with a non-significant change in CAT and GPX activities as compared to the HT-29 group. The exposure of HT-29 cells to IR caused insignificant changes in SOD and CAT activities, though a significant decrease (*p* < 0.05) in the levels of HIF-1α (30.77%), intracellular H_2_O_2_ (60.75%), MDA (35.56%), GSH (35.76%), and GPX activity (28.64%) as well as the protein expression of p-ERK1/2 (75.00%) and p-JNK (51.02%) coupled with a marked elevation (*p* < 0.05) in NRF-2 level (1.89-fold) was observed as compared to the HT-29 group (Figure 14). Combining Eb-ZCFO with IR induced a significant reduction (*p* < 0.05) in HIF-1α (61.30%), intracellular H_2_O_2_ (62.88%), MDA (64.54%), p-ERK1/2 (80.54%), and p-JNK (58.07%) and a significant increase (*p* < 0.05) in SOD activity (1.39-fold) and NRF-2 level (2.02-fold) compared with the HT-29 group (Figure 14). A delicate balance of the intracellular ROS levels is essential for cancer cells. High levels of ROS encourage tumour development and progression. Thus, the fine-tuning of intracellular ROS signalling is a challenge for novel therapeutic strategies. This, achieved through depriving cells of ROS-sensing signalling pathways, induces tumour progression, versus tipping the balance to ROS-induced apoptotic signalling [73]. In many types of cancers, ROS-sensitive signalling pathways such as mitogen-activated protein kinase/ERK cascade, signal transducer and activator of transcription (STAT), and nuclear factor κ-B (NF-κB)-activating pathways are elevated and participate in cell proliferation, regulate protein synthesis and activity, induce inflammation, and promote cell survival [73,74]. In response to inflammatory signals, interferon gamma (IFN-γ) and lipopolysaccharide (LPS) sensitised macrophages polarise into the classical or “M1” state, which is characterised by the secretion of pro-inflammatory signals, such as tumour necrosis factor-alpha (TNF-α), interleukin 6 (IL-6), and IL-12. In contrast, alternatively activated macrophages, known as M2 macrophages, are polarised by anti-inflammatory signals such as IL-4, IL-10, and IL-13 [75]. Ohmori and Hamilton [76] and Hobson-Gutierrez and Carmona-Fontaine [75] demonstrated that the JAK-STAT pathway is an essential part of the pro-inflammatory (via STAT-1, which is activated by IFN-γ) and anti-inflammatory (via STAT-6, which is activated by IL-4) responses that generate “M1” and “M2” macrophages, respectively. The health of tissues and the quality of the cellular compartments are actively maintained by a range of cell–cell interactions, in a process known as cell competition. Through cell competition, cells sense fitness level heterogeneities across cell populations, resulting in the elimination of the less-fit cells (or losers) when they are in the presence of fitter cells (or winners), in a process akin to natural selection. It could be postulated that cell competition for healthy life is present between normal (winners) and cancers (losers) in the TME. The stress response pathways, including the Jun N-terminal kinase (JNK), STAT, and the transcription factor NRF-2 that targets many genes involved in the oxidative stress response pathways, play vital roles in cellular competition and induce the three main aspects of the competition process: slow proliferation of losers, over-proliferation of winners, and loser cell elimination [77]. Owing to the complexity of ROS interconnections with stress-sensing signalling pathways, tight regulation and fine-tuning of intracellular ROS and scavengers is a recent approach in cancer therapy [78]. Cancer-associated fibroblasts (CAFs), which are highly represented in the TME, actively contribute to the regulation of tumour homeostasis, the promotion of tumour progression, and the invasion of cancer cells [79]. ROS and CAFs participate in two-way crosstalk: on the one hand, fibroblasts are targeted by ROS, particularly H_2_O_2_, which is able to transform them into active CAFs via the upregulation of HIF1α. On the other hand, CAFs are critical for the increase in ROS levels observed in cancer [80]. ROS largely impacts the redox-sensitive kinases ERK1/2 and JNK and induces proto-oncogenes [81,82]. Upon phosphorylation of the target proteins, these kinases can induce cell proliferation and survival [81]. Tumour cells have more abundant ROS levels than their normal counterparts, which are responsible for the maintenance of the malignant phenotype. Tenacious ROS stress enables cancer cells to adapt and survive [83]. Therefore, utilising drugs that decrease ROS and enhance the antioxidant machinery in tumour cells overwhelms the stress-sensing signalling pathways and renders them more vulnerable to external stimuli, especially when combined with therapies that elevate ROS, such as γ-radiation, as seen in our data. The antitumour efficiency of the ZFO and ZCFO nano-platform could be attributed to Eb, which blocked ADAM-9, a disintegrin and metalloprotease-9, by inhibiting ROS production, and reduced the progression of human prostate cancer cells [84]. Gao et al. [85] and Kang et al. (2019) [86] found that the expression of apoptosis-related proteins and antioxidant enzymes was increased through the upregulation of NRF-2 in cancer cells. Kucinski et al. [77] found that NRF-2 overexpression abolishes JNK expression, indicating that NRF-2 is not upstream of constitutive JNK activation, and therefore JNK activation is not necessary for cells to acquire loser status. As well as Eb, zinc oxide nanoparticles (ZnO-NPs) are known for toxicity and the ability to form nanoparticle–cell contacts [87].

Walkey et al. [88] showed that nanoceria selectively protect normal cells, but not cancer cells, from damage; in cancer cells, nanoceria are pro-apoptotic. This selective toxicity of nanoceria against cancer cells is due to the inhibition of nanoceria catalase-like activity occurring in acidic (pH 4.3) environments, and this is based on the assumption that the pH of cancer microenvironment is low due to the Warburg effect. Das et al. [89] reported that Ce-NPs in the A2780 ovarian cancer cell line had decreased ROS generation compared with control. These researchers explored the pro-oxidant effect of Ce-NPs as radiation sensitisers for pancreatic cancer radiotherapy. Pešić et al. [90] suggested the potential of using Ce-NP as a treatment for colorectal carcinoma, as it would selectively eliminate cancer cells and leave healthy cells intact. Ce-NPs serve as an enduring redox metabolism regulator rather than as simple scavengers, efficiently eliminating ROS when needed and thus maintaining basal cellular activities. They could therefore be used as a bio-compatible antioxidant system [91]. Their ROS-scavenging properties make Ce-NPs an attractive countermeasure against the detrimental effects of IR on normal cells [92]. Owing to their unique ROS-scavenging properties, Ce-NPs offer a valuable tool to aid in achieving ROS fine-tuning within the TME and prevent IR-associated NRF-2 over activation. Rice et al. [93] reported that a diminished level of phosphorylated ERK-1/2-MAPK protein was detected after CeO_2_ instillation into rat lung. McDonald et al. [94] demonstrated that the MCF-7 cell line displayed a dose-dependent cytostatic, rather than cytotoxic, response to radiation, instead of rapid inter-phase death within hours by apoptosis like most cell lineages that exhibit mitotic cell death, autophagy, or senescence, and respond only over a period of many days, through the activation of NRF-2.

#### 2.6.3. Inflammatory Status and Crosstalk of Signalling Pathways TNF-α/NF-κB, INF-γ/STAT-1, and IL-4/STAT-6 in MDA-MB-231 and HT-29 Cell

##### MDA-MB-231 Cell Line

**Figure 15:**
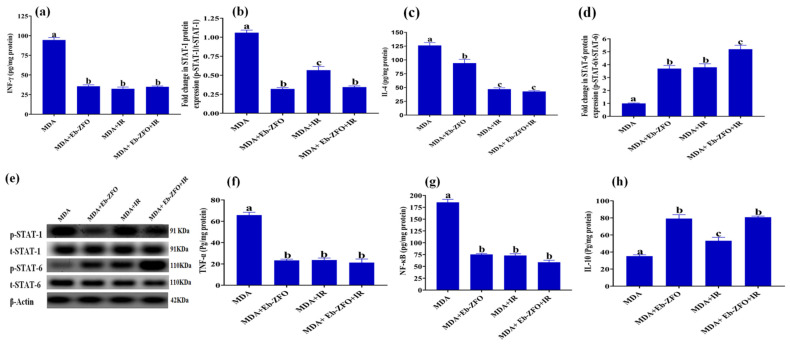
Inflammatory status and crosstalk of the signalling pathways TNF-α/NF-κB, INF-γ/STAT-1, and IL-4/STAT-6 in MDA-MB-231 cells. Represented as follows; levels of (**a**) INF-γ, (**b**) protein expression of STAT-1, (**c**) IL-4, (**d**) protein expression of STAT-6, (**e**) representative western blot analysis, SDS-PAGE of STAT-1, STAT-6, and β- actin, (**f**) TNF-α, (**g**) NF-κB and (**h**) IL-10. Results are expressed as the mean and standard error of the mean (SEM) (n = 3). Columns with dissimilar letters (a, b, c…) overhead within the same histogram are significantly different, and columns with the same letters are not significantly different at *p* < 0.05. MDA group: untreated MDA-MB-231 cells served as control, MDA + Eb-ZCFO group: MDA-MB-231 cells line treated with Ebselen (Eb) and ZnFe_2_O_4_ nanoparticles (ZFO), MDA + IR group: MDA-MB-231 cells exposed to γ-radiation, and MDA + Eb-ZFO + IR group: MDA-MB-231 cells treated with Eb-ZFO and exposed to IR.

The application of Eb-ZFO, IR, and Eb-ZFO + IR to MDA-MB-231 cells induced a significant decline (*p* < 0.05) in the inflammatory mediators NF-κB by 59.33%, 60.52%, and 68.28%; IL-4 by 25.41%, 62.71%, and 66.09%; INF-γ by 62.33%, 65.71%, and 63.07%; and TNF-α by 64.44%, 63.98%, and 67.63%, respectively. The protein expression of p-STAT-1 signalling effector declined by 69.80%, 46.54%, and 67.55%, respectively, linked with a significant increase (*p* < 0.05) in IL-10 level by 2.25-fold, 1.51-fold, and 2.29-fold, respectively, and p-STAT-6 protein expression by 3.697-fold, 3.799-fold, and 5.199-fold, respectively, as compared to the MDA group (Figure 15).

##### HT-29 Cell Line

**Figure 16:**
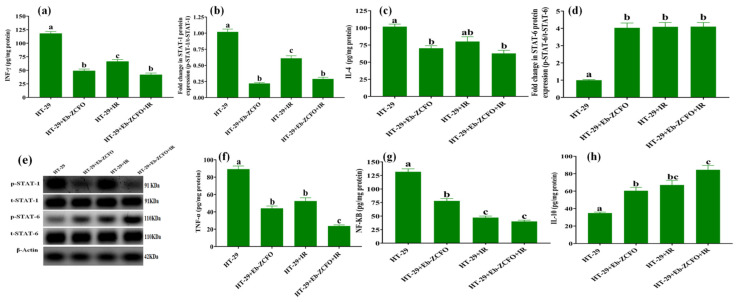
Inflammatory status and crosstalk of the signalling pathways TNF-α/NF-κB, INF-γ/STAT-1, and IL-4/STAT-6 in HT-29 cells. Represented as follows: levels of (**a**) INF-γ, (**b**) protein expression of STAT-1, (**c**) IL-4, (**d**) protein expression of STAT-6, (**e**) representative western blot analysis, SDS-PAGE of STAT-1, STAT-6, and β- actin, (**f**) TNF-α, (**g**) NF-κB, and (**h**) IL-10. Results are expressed as the mean and standard error of the mean (SEM) (n = 3). Columns with dissimilar letters (a, b, c…) overhead within the same histogram are significantly different, and columns with the same letters are not significantly different at *p* < 0.05. HT-29 group: untreated HT-29 cells line served as control, HT-29 + Eb-ZCFO group: HT-29 cells line treated with Ebselen (Eb) and ZnCe_0.06_Fe_1.94_O_4_ nanoparticles (ZCFO), HT-29 + IR group: HT-29 cells exposed to γ-radiation, and HT-29 + Eb-ZCFO + IR group: HT-29 cells treated with Eb-ZCFO and exposed to IR.

Treatment of HT-29 cells with either Eb-ZCFO or IR exposure or both Eb-ZCFO + IR showed a significant decrease (*p* < 0.05) in the inflammatory mediators NF-κB 40.71%, 64.10%, and 69.49%; IL-4 30.90%, 21.31%, and 38.37%; INF-γ 58.29%, 43.82%, and 64.38%; and TNF-α 50.56%, 41.14%, and 73.43%, and the protein expression of STAT-1 by 78.41%, 40.18%, and 71.55%; signalling effector showed a significant rise (*p* < 0.05) in IL-10 level of 1.73-fold, 1.92-fold, and 2.42-fold; and its signalling effector STAT-6 protein expression by 4.03-fold, 4.09-fold, and 4.1-fold, respectively, as compared to the HT-29 group (Figure 16). The link between chronic inflammation and tumour development is well-established, and it has become evident that an inflammatory microenvironment is a prerequisite for all tumours, including those that emerge in the absence of overt inflammation [95]. Chronic inflammation affects the TME and impacts cell plasticity through the epithelial–mesenchymal transition, dedifferentiation, the polarisation of immune cells, production of ROS and cytokines, epigenetic mechanisms, miRNAs, and complex regulatory cascades in tumour and stromal cells [96]. The current data showed a considerable reduction in pro-inflammatory TME through the activation of STAT-6 expression. Consistently with our data, a study by Ohmori and Hamilton [76] found that STAT-6 mediates the suppression of INF-γ/STAT-1 and TNF-α/NF-κB-dependent transcription by distinct mechanisms. Park et al. (2019) [97] found that the restoration of STAT-6 levels in glioblastoma (GBM) suppresses HIF-1α protein synthesis and induces STAT-6-regulated immune responses and apoptosis, thereby leading to the suppression of GBM proliferation. Tissue hypoxia in cancer induces cell growth, neovascularisation, invasion, resistance to chemo and radiotherapy, and ultimately recurrence after treatment [97]. IL-10 is a potentially valuable target of immunomodulatory therapy, as observed by Brunn et al. [98], who postulated a dual role for macrophages during initiation and recovery from experimental autoimmune neuritis (EAN), especially in the imbalance between autoimmune pro-inflammatory milieu and the net effect of various immunoregulatory mediators, such as IL-10, IL-4, and STAT-6. It was found that the absence of a single anti-inflammatory Th2-derived cytokine, such as IL-10 or IL-4, and even the absence of a single downstream signalling molecule of the IL-4 pathway, STAT6, markedly interferes with recovery from EAN. Tewari et al. [99] found that Eb not only down-regulated the enhanced ROS production of TNF-α treated glioma cells but also overcame TNF-α-induced pro-inflammatory mediators to prevent the build-up of a deleterious pro-inflammatory TME. Based on the current data, Eb appears to efficiently modulate the pro-inflammatory TME via augmentation of STAT-6 expression and its antioxidant and immunoregulatory activity. Irradiation was found to promote M2 phenotype macrophages in hypoxic TME, thereby directing the pro-inflammatory milieu within tumours toward an alternative anti-inflammatory TME [100]. Thabet and Moustafa [101] found that Eb and γ-radiation at 1, 3, and 6 Gy induced apoptosis and anti-angiogenic and antiproliferative effects by reducing NF-κB signalling and increasing IL-10 in MCF-7 cells. One of the factors contributing to the anti-inflammatory effect of IR in the current study is NRF-2 upregulation in response to irradiation. Considering the biphasic role of NRF-2 in cancer therapy [102], constitutive activation of NRF-2 is associated with the promotion of development of several cancers, poor diagnosis in clinical settings, and resistance to therapies [103]. NRF-2 hyperactivity in cancer cells confers chemo- and radioresistant characteristics [102]. However, activation of the Nrf2/ARE signalling pathway plays a critical role in the alleviation of chronic inflammation, which is associated with cancers, since Nrf2 positively regulates a large number of cytoprotective proteins. Elimination of ROS has been widely accepted as the molecular basis of Nrf2-mediated anti-inflammation [104,105]. Hence, the boost in NRF-2 and STAT-6 expression elicited by Eb-ZCFO or IR exposure, or their combination, produces an anti-inflammatory response within the TME that might hinder tumour progression, as revealed in the present study.

#### 2.6.4. Assessment of Autophagy and Apoptosis-Related Proteins in MDA-MB-231 and HT-29 Cells

##### MDA-MB-231 Cell Line

**Figure 17:**
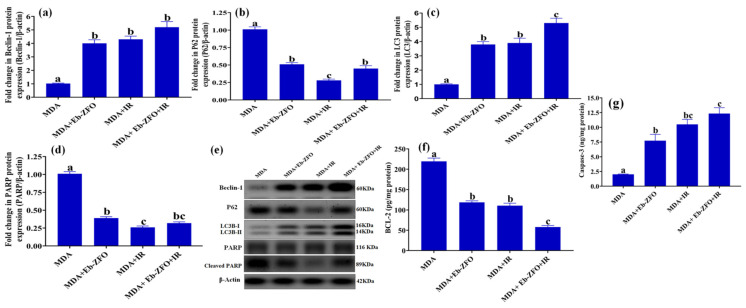
Autophagy and apoptosis in MDA-MB-231 cells. Represented as follows: fold change in protein expression of (**a**) beclin-1, (**b**) P62, (**c**) LC3B-II, (**d**) cleaved PARP-1, (**e**) representative western blot analysis, SDS-PAGE of beclin-1, P62, LC3B-II/LC3B-I, cleaved PARP-1 and β-actin, (**f**) BCL-2, and (**g**) cleaved caspase-3 protein levels. Results are expressed as the mean and standard error of the mean (SEM) (n = 3). Columns with dissimilar letters (a, b, c…) overhead within the same histogram are significantly different, and columns with the same letters are not significantly different at *p* < 0.05. MDA group: untreated MDA-MB-231 cells served as control; MDA + Eb-ZFO group: MDA-MB-231 cells treated with Ebselen (Eb) and ZnFe_2_O_4_ nanoparticles (ZFO); MDA + IR group: MDA-MB-231 cells exposed to γ-radiation; and MDA + Eb-ZFO + IR group: MDA-MB-231 cells treated with Eb-ZFO and exposed to IR.

The data shown in Figure 17 show a significant increase (*p* < 0.05) in autophagy markers (Beclin-1 3.92-fold, 4.21-fold, and 5.09-fold and LC3B-II/I 3.8-fold, 3.9-fold, and 5.3-fold) and apoptosis-related protein cleaved caspase-3 (3.85-fold, 5.23-fold, and 6.14-fold) associated with a significant diminishment (*p* < 0.05) in p62 (49.52%, 72.27%, and 55.46%), caspase-dependent cleaved poly (ADP-ribose) polymerase (PARP), an apoptosis marker and enzyme responsible for DNA repair (61.39%, 74.26%, and 68.35%) and anti-apoptotic B-cell lymphoma 2 (BCL-2) (45.87%, 49.66%, and 73.64%) in MDA-MB-231 cells treated with Eb-ZFO, IR, and Eb-ZFO + IR, respectively, when compared to the MDA group (Figure 17).

##### HT-29 Cell Line

**Figure 18:**
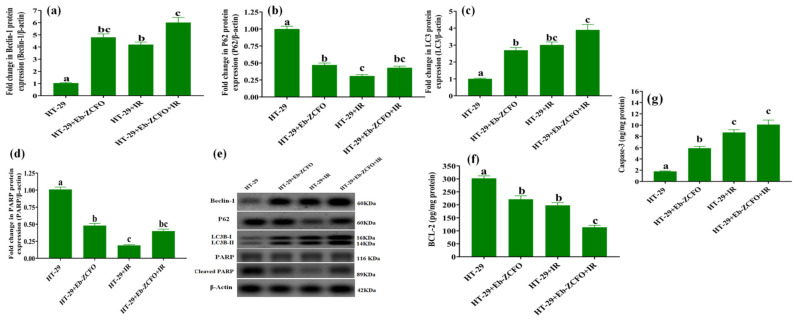
Autophagy and apoptosis in HT-29 cells. Represented as follows: fold change in protein expression of (**a**) Beclin-1; (**b**) P62; (**c**) LC3B-II; (**d**) cleaved PARP-1; (**e**) representative western blot analysis, SDS-PAGE of Beclin-1, P62, LC3B-II/LC3B-I, cleaved PARP-1, and β-actin; (**f**) BCL-2; and (**g**) cleavedcaspase-3 protein levels. Results are expressed as the mean and standard error of the mean (SEM) (n = 3). Columns with dissimilar letters (a, b, c…) overhead within the same histogram are significantly different, and columns with the same letters are not significantly different at *p* < 0.05. HT-29 group: untreated HT-29 cells served as control. HT-29 + Eb-ZCFO group: HT-29 cells treated with Ebselen (Eb) and ZnCe_0.06_Fe_1.94_O_4_ nanoparticles (ZCFO). HT-29 + IR group: HT-29 cells exposed to γ-radiation. HT-29 + Eb-ZCFO + IR group: HT-29 cells treated with Eb-ZCFO and exposed to IR.

A significant increase (*p* < 0.05) was observed in the expression of autophagy proteins (Beclin-1 4.62-fold, 4.04-fold, and 5.78-fold and LC3B-II/I 2.67-fold, 2.98-fold, and 3.86-fold) and apoptosis-related protein; cleaved caspase-3 (3.27-fold, 4.83-fold, and 5.61-fold) was observed in HT-29 cells treated with Eb-ZCFO or exposed to IR or both (Figure 18). Eb-ZCFO + IR groups associated with a significant reduction (*p* < 0.05) in p62 (52.93%, 69.00%, and 57.00%) and cleaved PARP protein expression (52.46%, 81.18%, and 60.39%) as well as the anti-apoptosis protein; BCL-2 level (26.61%, 34.39%, and 62.39%), respectively, as compared to the HT-29 group (Figure 18). Autophagy is a recently recognised response of tumour cells to various anticancer therapies, including radiotherapy and chemotherapy [106]. It is a highly conserved cellular catabolic process that degrades and recycles cellular components through lysosomes [107]. During starvation, cells can supply self-nutrition via lysosomal enzyme-induced degradation of macromolecules and damaged proteins [108]. Autophagy, however, can serve as a double-edged sword and activate cellular apoptosis through type II programmed cell death. Through the interaction between the C-terminal cleavage of light chain 3 (LC3)-associated microtubule protein (LC3B peptide) and autophagy-related gene 4, which is a type of cysteine protease, autophagy is induced. It produces LC3BI, which conjugates with phosphatidylethanolamine (PE) to yield LC3BII [109], which can integrate with autophagy-related proteins 5, 7, and 12 to create autophagosomes, along with phospholipid bilayers. In contrast, LC3 interacts with p62/sequestome-1 (SQSTM1), which functions as a ubiquitin-binding protein to break down damaged organelles and macromolecules [110]. Therefore, the levels of proteins LC3 and p62 are broadly recognised as prominent markers of autophagy [111,112]. As shown in our data, an increase in the autophagic flux was detected in MDA-MB-231 and HT-29 cells, as indicated by enhancedbeclin-1 and LC3BII protein expression, paralleled by boosted cleaved caspase-3 along with reduced BCL-2 and p62 protein levels, suggesting a switch from cytoprotective to cytotoxic autophagy, which resulted in apoptotic death induced by Eb-ZCFO or IR, or their combination. Ample evidence has shown an association between the induction of autophagy and radioresistance in a wide range of tumours, including pancreatic cancer, breast carcinoma, and glioma [113,114,115].

The antitumour effect of Beclin-1 has been confirmed in many types of tumours such as breast [116], colon [117], cervical [118], ovarian [119], and glioblastoma [120]. Huang et al. (2014) [121] found that the autophagy gene Beclin-1 promotes apoptosis and reduces cell proliferation by increasing the expression of LC3 and caspase-3, associated with a decreased expression of p62 and Bcl-2. Thus, Beclin-1 plays an important role in the fine-tuning of autophagy and apoptosis. Song et al. [122] found that AKT inactivation-induced elevation of Beclin-1 cleavage resulted in disruption of the R-BiP/Beclin-1/p62 complex, which led to switching from autophagy to the synergistic induction of apoptosis. Baek et al. [123] found that Eb decreased the phosphorylation of IκB, PI3K, and AKT in lipopolysaccharide-induced inflammatory bone destruction models. A study by Kaczor-Keller et al. [20] demonstrated that Eb efficiently inhibits cancer cell proliferation, induces G2/M cell-cycle arrest, and promotes cell death in prostate cancer by switching from apoptotic cell death to necrosis via a significant decrease in the level of cleaved PARP after 24 h exposure to Eb. Although necrosis as an uncontrolled modality of cell death is generally associated with damage to peripheral tissues and increased systemic inflammation, recent observations highlight a positive role for necrosis induction during cancer therapy [20]. PARP-1 plays an important role in the response to IR-induced DNA damage and may confer radioresistance [124]. Hampering PARP-1 activity may therefore be a successful approach to the sensitisation of TNBC and colorectal adenocarcinoma to radiotherapy, a process that was achieved by Eb-ZFO and Eb-ZnCe_0.06_Fe_1.94_O_4_ nanocomposites in our study.

#### 2.6.5. Flow Cytometric Analysis of Cell Cycle inMDA-MB-231 and HT-29 Cells

The flow cytometric analysis of the cell-cycle distribution was used to study the anticancer effects of Eb + ZnFe_2_O_4_ (Eb-ZFO) on MDA-MB-231 cells and Eb + ZnCe_0.06_Fe_1.94_O_4_ (Eb-ZCFO) on HT-29 cells alone and in combination with γ-radiation (IR; 4 Gy) for 24 h.

##### MDA-MB-231 Cell Line

**Figure 19:**
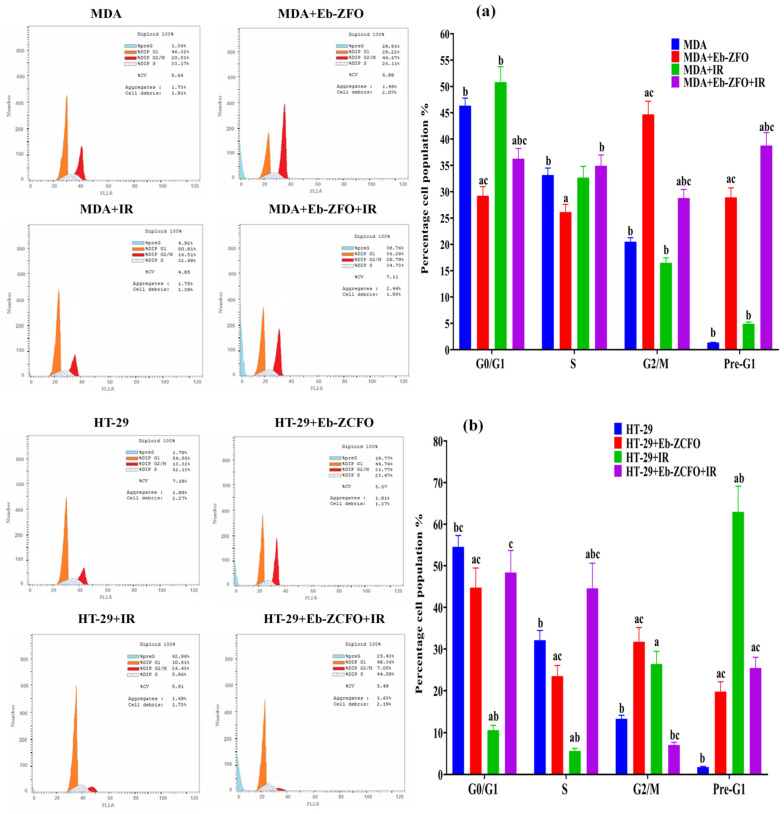
Cell-cycle analysis via flow cytometry. (**a**) Distribution of MDA-MB-231cells through the cell-cycle phases after treatment with Eb-ZFO alone or in combination with 4 Gy γ-radiation for 24 h. MDA group: untreated MDA-MB-231 cells served as control. MDA + Eb-ZFO group: MDA-MB-231 cells treated with Ebselen (Eb) doped intoZnFe_2_O_4_nanoparticles (ZFO). MDA + IR group: MDA-MB-231 cells exposed to γ-radiation. MDA + Eb-ZCFO + IR group: MDA-MB-231 cells treated with Eb-ZFO and exposed to IR. (**b**) Distribution of HT-29 cells through the cell-cycle phases after treatment with Eb-ZCFO alone or in combination with 4 Gy γ-radiation for 24 h. HT-29 group: untreated HT-29 cells served as control. HT-29 + Eb-ZCFO group: HT-29 cells treated with Ebselen (Eb) and ZnCe_0.06_Fe_1.94_O_4_ nanoparticles (ZCFO). HT-29 + IR group: HT-29 cells exposed to γ-radiation. HT-29 + Eb-ZCFO + IR group: HT-29 cells treated with Eb-ZCFO and exposed to IR. Results are expressed as the mean and standard error of the mean (SEM) (n = 3). Columns with a, b, and c letters overhead within the same phase are significantly different from the control, treated group, or IR group of each cell line, respectively, at *p* < 0.05.

The data from the MDA + Eb-ZFO group showed a significant decrease (*p* < 0.05) in the percentage of cells in the G0/G1and S phases, associated with a noticeable accumulation of cells in the G2/M and pre-G1 phases. This distribution caused cell-cycle arrest at the G2/M phase as compared to the MDA group (Figure 19a). After exposure to γ-radiation, the MDA + Eb-ZFO + IR group showed a significant decrease (*p* < 0.05) in the percentage of cells in G0/G1 phase, associated with an accumulation of cells in the G2/M and pre-G1 phases, causing cell-cycle arrest at the pre-G1phase as compared to the MDA group (Figure 19a).

##### HT-29 Cell Lines

The data from the HT-29 + Eb-ZCFO group showed a significant decrease (*p* < 0.05) in the percentage of cells in the G0/G1 and S phases associated with an accumulation of cells in the G2/M- and pre-G1 phases, causing cell-cycle arrest at the G2/M phase as compared to the HT-29 group (Figure 19b). After exposure to γ-radiation, the results from the HT-29 + Eb-ZCFO + IR group displayed a significant increase in S- and pre-G1 phases and caused cell-cycle arrest at the S-phase, compared with the HT-29 group (Figure 19b). The cell cycle includes a number of checkpoints that allow the cell to repair its damaged DNA. Checkpoints at the G1/S and G2/M transitions are essential regulatory gates during cell-cycle progression, whereas loss of cell-cycle checkpoints ahead of completing DNA repair can activate the apoptotic cascade and result in cell death [125]. Our data indicated that Eb-ZFO and Eb-ZCFO induced cell-cycle arrest at the G2/M phase in both MDA-MB-231 and HT-29 cells, suggesting that cells undergo apoptosis or mitotic catastrophe, but when exposed to IR, as observed in the MDA + Eb-ZFO + IR and HT-29 + Eb-ZCFO + IR groups, caused cell-cycle arrest at the sub-G1 (total apoptosis) and S-phases, respectively, suggesting the fragmentation of DNA and impairment of DNA synthesis and replication. Most antitumour agents interrupt the cell-cycle checkpoints at the G0/G1, S, and G2/M phases and then trigger apoptosis [126]. The G2 checkpoint prevent cells from entering mitosis when the DNA is damaged, providing an opportunity for repair and prevention of the proliferation of damaged cells [127]. Cytotoxic drugs that cause S-phase arrest prevent accurate DNA synthesis and replication [128].

#### 2.6.6. Detection of Apoptosis in MDA-MB-231 and HT-29 Cells Lines by Flow Cytometry

Apoptosis and necrotic cell death were assessed using flow cytometric analysis with Annexin-V-FITC and PI dual-staining to evaluate the pro-apoptotic effects of Eb + ZnFe_2_O_4_ (Eb-ZFO) on MDA-MB-231 cells and Eb + ZnCe_0.06_Fe_1.94_O_4_ (Eb-ZCFO) on HT-29 cells, alone and in combination with γ-radiation (IR; 4 Gy) for 24 h.

##### MDA-MB-231 Cell Lines

**Figure 20:**
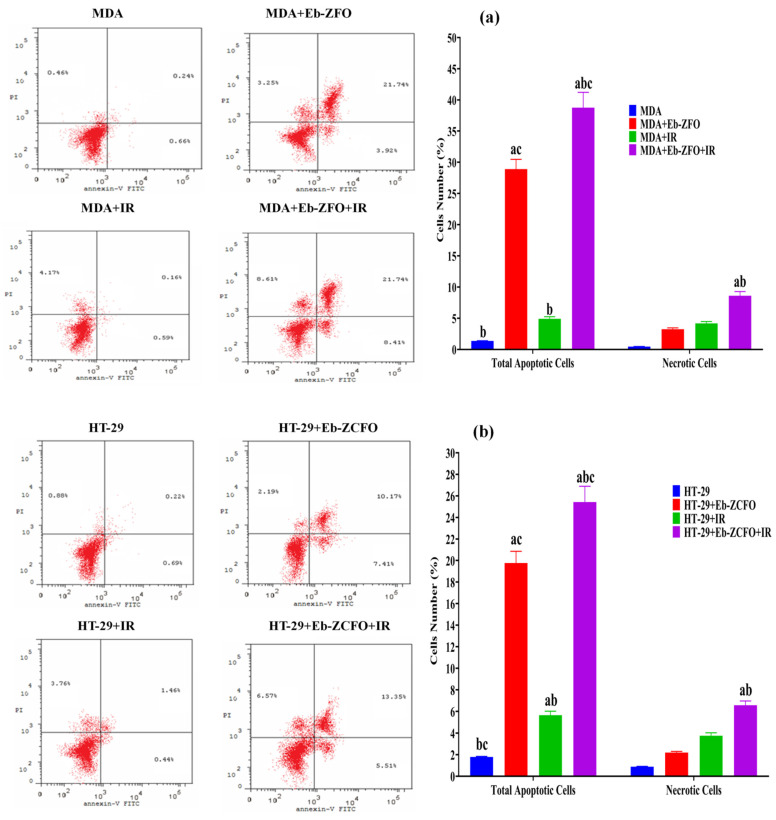
Detection of apoptosis via flow cytometry. (**a**) Eb-ZFO and IR induced apoptosis and necrosis in MDA-MB-231 cells after 24 h. The upper-left panel shows representative images of cell apoptosis. The percentage of apoptotic and necrotic cells was determined and is represented in the upper-right panel. MDA group: untreated MDA-MB-231 cells line served as control. MDA + Eb-ZFO group: MDA-MB-231 cells treated with Ebselen (Eb) and ZnFe_2_O_4_ nanoparticles (ZFO). MDA + IR group: MDA-MB-231 cells exposed to γ-radiation. MDA + Eb-ZFO + IR group: MDA-MB-231 cells treated with Eb-ZFO and exposed to IR. (**b**) Eb-ZCFO and IR induced apoptosis and necrosis in HT-29 cells after 24 h. HT-29 group: untreated HT-29 cells served as control. HT-29 + Eb-ZCFO group: HT-29 cells treated with Ebselen (Eb) and ZnCe_0.06_Fe_1.94_O_4_ nanoparticles (ZCFO). HT-29 + IR group: HT-29 cells exposed to γ-radiation. HT-29 + Eb-ZCFO + IR group: HT-29 cells treated with Eb-ZCFO and exposed to IR. The lower-left panel shows representative images of cell apoptosis. The percentage of apoptotic and necrotic cells was determined and is shown in the lower-right panel. The results are expressed as the mean and standard error of the mean (SEM) (n = 3). Columns with a, b, and c letters overhead within the same phase are significantly different from control, treated group, or IR group of each cell line at *p* < 0.05.

The proportion of apoptotic and necrotic cells in a population of MDA-MB-231 cells treated with IC_50_ is shown in Figure 20a. In Figure 20a, the data from the MDA + Eb-ZFO and MDA + Eb-ZFO + IR groups show a significant increase (*p* < 0.05) in the total apoptotic cells (early and late) compared to the MDA group. The MDA + Eb-ZFO + IR group showed a significant increase (*p* < 0.05) in necrotic cells compared to the MDA, MDA + Eb-ZFO, and MDA + IR groups. Based on these observations, it could be concluded that γ-radiation (IR) elevates both apoptotic and necrotic death in TNBC cells when coupled with Eb-ZFO nanocomposite (Figure 20a).

##### HT-29 Cell Lines

Figure 20b shows data from the HT-29 + Eb-ZCFO, HT-29 + IR, and HT-29 + Eb-ZCFO + IR groups, revealing a significant increase (*p* < 0.05) in the total apoptotic cells (early and late) compared to the MDA group. The HT-29 + Eb-ZCFO + IR group showed a significant elevation (*p* < 0.05) in necrotic cells compared to the HT-29 and HT-29 + Eb-ZCFO groups. Accordingly, it could be concluded that γ-radiation (IR) promotes apoptotic and necrotic death in colorectal adenocarcinoma cells when combined with Eb-ZCFO nanocomposites (Figure 20b). A complex relationship is evident between autophagy and apoptosis. Autophagy frequently occurs with or before apoptosis. In the latter case, a surge in autophagic flux regulates tumour cell growth by facilitating the induction of apoptosis or necrosis [129,130]. As a crucial cellular process, apoptosis is modulated by multiple regulatory molecules and different pathways [131]. These modulators are essential for regulating the growth of various cancers. Both the blockage of pro-apoptotic Bcl-2 family molecules and the enhancement of anti-apoptotic family signals are required for the modulation of apoptotic dysregulation in tumours [132]. Caspases, a family of cysteine acid proteases, are key regulators of cell survival and apoptosis [133]. Our findings indicated that Eb-ZFO or Eb-ZCFO alone or in combination with IR could reduce the level of BCL-2 protein, which is known to be an essential anti-apoptotic signal in MDA-MB-231 and HT-29 cells, accompanied by an increase in cleaved caspase-3 protein, coupled with a marked curtailment of PARP-1 cleavage. In a mouse proximal tubular cell ATP depletion model, Lieberthal et al. [134] found that mild ATP depletion led to apoptosis, whereas severe ATP depletion led to necrosis. ATP is required for apoptosis, such that severe ATP depletion inhibits the apoptotic pathway in a number of cell types [135,136]. This phenomenon might explain the dual induction of apoptotic and necrotic cells in Eb-ZFO and Eb-ZCFO nanocomposites alone or when combined with IR, suggesting the radiosensitisation of MDA-MB-231 and HT-29 cells. A marked increase in the population of necrotic cells in the MDA-Eb-ZFO + IR and HT-29 + Eb-ZCFO + IR groups suggests the strong inhibition of ATP with the activation of ATP-independent necrosis.

**Figure 21:**
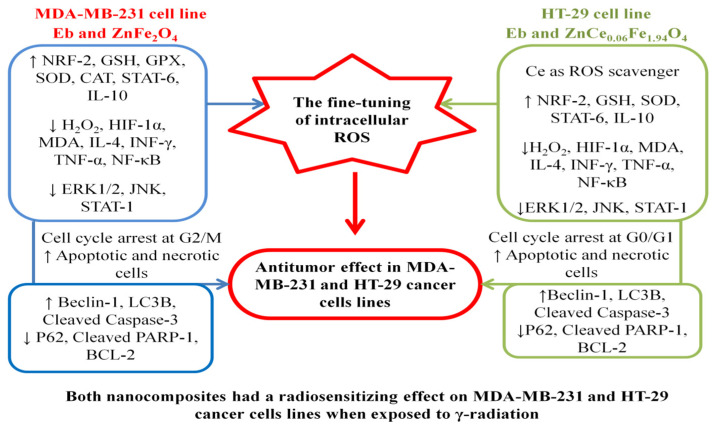
Signalling pathways involved in the antitumour activity of Eb + ZnFe_2_O_4_ and ZnCe_x_Fe_2−x_O_4_; X = 0.06 nanocomposites, where the effect is mediated through the antioxidant, anti-inflammatory, and immunomodulatory effects of Eb, plus the cytotoxicity of ZnO-NPs in MDA-MB-231cells, whereas the cytotoxic effect of ZnCe_0.06_Fe_1.94_O_4_ relies mainly on Ce^3+^ as a ROS scavenger and the immunomodulatory capability of Eb in HT-29 cells, leading to an overwhelming response in the tumour antioxidant machinery, coupled with reversion of the pro-inflammatory TME to an anti-inflammatory milieu. This reversion, in turn, resulted in an alteration in the tumour proliferation pathways, accompanied by a switch from cytoprotective to cytotoxic autophagy, and eventually a marked rise in pro-apoptotic and decrease in anti-apoptotic proteins. Treatment of both cells with Eb-ZFO or Eb-ZCFO alone or in combination with IR provoked cell-cycle arrest at the major checkpoints, with elevated levels of apoptotic and necrotic cells, suggesting an excellent antitumour effect and radiosensitisation of TNBC and colorectal adenocarcinoma cells.

Overall, as shown in Figure 21, the mechanism of Eb-ZFO in the MDA-MB-231 cell line depends primarily on the Eb antioxidant/anti-inflammatory capability and the cytotoxicity profile of the ZnO-NPs. However, the exhibited antitumour activity of Eb-ZCFO in HT-29 cell line depends on Ce nanoparticles as ROS scavenger and Eb as immunomodulatory of TME. The findings of the current study agree with those obtained by Barrera et al. [137] and Liu et al. [138].

## 3. Materials and Methods

### 3.1. Synthesis of ZnCe_x_Fe_2−x_O_4_ Nanoferrites

The (Ce (NO_3_)_3_.6H_2_O, 99.99%), (Fe (NO_3_)_3_·9H_2_O, 98.0%), (ZnSO_4_.7H_2_O, 98%), (C_6_H_8_O_7_, 99.57%), and (C_2_H_6_O_2_, 99.8%) were purchased from Sigma-Aldrich, Germany and used as received without further purification. The synthesis of ZnCe_x_Fe_2−x_O_4_ (X = 0.0, 0.02, 0.04, 0.06, 0.08) powders was carried out using a facile sol–gel method as described in detail in our previous work [10,11,12,25,34,35].

### 3.2. Characterisation of the Nanoferrites

The stoichiometry of the ZnCe_x_Fe_2−x_O_4_ (X = 0.0, 0.02, 0.04, 0.06, 0.08) samples was examined using energy dispersive X-ray spectra (EDX) (JEOL JSM-5600 LV, Japan). In order to confirm the formation of the spinel ferrite phase, Fourier transform infrared (FTIR) spectroscopy using a NICOLET iS10 model instrument was conducted over a range from 350 to 3000 cm^−1^. The crystal structure of the samples was investigated using X-ray diffraction (XRD) (Shimadzu XRD-6000, Japan). XRD patterns were obtained in a range of 2θ from 17° to 90° at room temperature. Cu Kα was used as the radiation source of wavelength λ = 0.15408 nm, with a scan rate of 0.8°/min, an operating voltage of 50 kV, and a current of 40 mA [139,140]. Information about the shape and grain size of the sample particles was obtained using high resolution scanning electron microscopy (SEM) (JEOL JSM-5600 LV, Japan). Finally, the particle size distribution, the hydrodynamic radius, and the polydispersity index (PDI) of the synthesised ZnCe_x_Fe_2−x_O_4_ (X = 0.0, 0.02, 0.04, 0.06, 0.08) samples were determined by dynamic light scattering (DLS; Malvern devise, UK) and the indirect measurement of the surface charges of ZnCe_x_Fe_2−x_O_4_ (X = 0.0, 0.02, 0.04, 0.06, 0.08) samples was estimated by the zeta potential analyser (Malvern devise, UK) at the culture media as used in the treatments.

### 3.3. Antimicrobial Activities of ZnCe_x_Fe_2−x_O_4_ Nanoparticles and Ebselen

The antimicrobial potential of the as-synthesised Zn ferrites and ferrites substituted with Ce and Ebselen against different pathogenic microbes, both yeast and bacteria, were examined using the agar-disc diffusion method [141].

The as-synthesised ZnCe_x_Fe_2−x_O_4_ nanoparticles (X = 0.0, 0.02, 0.04, 0.06, and 0.08) and Ebselen were dissolved in DMSO at a concentration of 0.01 mg/mL, equivalent to 10 ppm.

The synthesised nanocomposite powder must be dispersed into solvent (DMSO) and applied as solution to be diffused on the surface of the agar plate, so they have single nanoparticles rather than agglomerated nanoparticles. The activity of the as-synthesised compounds were examined against Gram-positive bacteria *(Staphylococcus aureus* ATCC 25923, *Proteus vulgaris* ATCC 26325, and *Proteus mirabilis* ATCC 26659*)* and Gram-negative bacteria *(Pseudomonas aeruginosa* ATCC 27853*, Escherichia coli* ATCC 25922, *Klebsiella pneumoniae* ATCC 28896, and *Salmonella typhi* ATCC 26510). All of the above bacteria were established and fixed from 2 to 5 × 10^8^ CFU/mL (0.5 McFarland; at 600 nm). The inhibition of bacterial growth was defined by the zone of inhibition (ZOI) after 24 h of incubation.

The antifungal potential of the as-synthesised ZnCe_x_Fe_2−x_O_4_ nanoparticles (X = 0.0, 0.02, 0.04, 0.06, and 0.08) and Ebselen against the pathogenic unicellular fungi *Candida albicans* ATCC 90028, and *Candida tropicalis* ATCC 90159 was also examined. The inoculums of the yeast cells were set from 1 to 4 × 10^7^ CFU/mL. Nystatin (NS) and amoxicillin (AX) were used as standard antibiotics. AX is similar to penicillin in its bactericidal action against susceptible bacteria during the stage of active multiplication. It acts through the inhibition of cell wall biosynthesis that leads to the death of the bacteria [142]. NS is an antifungal that is both fungistatic and fungicidal in vitro against a wide-variety of yeasts and yeast-like fungi. It exerts its antifungal effects via disruption of the fungal cell membrane [143].

The investigation of minimum inhibitory concentrations (MIC) was performed using a serially diluted Luria–Bertani (LB) broth of the synthesised NPs. A positive control consisting of the microorganism and the nutrient, a negative control consisting of the nutrient alone, and the ZnCe_x_Fe_2−x_O_4_ nanoparticles (X = 0.0, 0.02, 0.04, 0.06, and 0.08) and Ebselen (beginning with 0.1 mg/mL concentration; 100 ppm) were used. The MIC was defined following 24 h of incubation at 37 °C. The inocula of the bacteria were 3–5 × 10^8^ CFU/mL and 2–3 × 10^7^ CFU/mL for *Candida* species. The MICs were defined using enzyme-linked immunosorbent assays (ELISA) plates at 600 nm.

### 3.4. Antibiofilm Activities of ZnCe_x_Fe_2−x_O_4_ Nanoparticles (X = 0.0 and 0.06)

Qualitative measurement of biofilm inhibition was carried out as described by Christensen et al. [144]. The extent of the biofilm that formed at the tube wall in the absence and presence of the synthesised ZnCe_x_Fe_2−x_O_4_ nanoparticles was measured. The antibiofilm activity of the as-synthesised ZnCe_x_Fe_2−x_O_4_ nanoparticles at 10.0 µg/mL was measured in the selected bacteria and *Candida* spp. and compared with the non-treated control.

Five millilitres of the nutrient broth medium was added to each tube, and the bacteria and yeast were inoculated after adjusting 0.5 McFarland to 1–2.5 × 10^8^ CFU/mL. The tubes were incubated at 37.0 ± 0.5 °C for 24 h. The contents of the control and treated tubes were removed, mixed with phosphate buffered saline (PBS; pH 7.0), and desiccated. The bacterial and yeast cells that adhered to the tube walls were fixed with 5 mL sodium acetate (3.0%) for about 15 min and then rinsed with de-ionised water.

The biofilms that developed inside the tubes were stained with 15 mL of 0.1% crystal violet (CV) and washed with de-ionised water to remove the rest of the CV. For the semi-quantitative estimation of the antibiofilm activity, 5 mL of absolute ethanol was added to dissolve the stained bacterial and yeast biofilms. The optical density (O.D.) of the stained bacterial and yeast biofilms with CV was examined using a UV-Vis spectrophotometer at 570.0 nm. The percentage of inhibition bacterial and yeast biofilms was estimated using Equation (1):

Biofilm inhibition % = (O.D._Control sample_ − O.D._treated sample_)/O.D._Control sample_ × 100 (1)

### 3.5. Reaction Mechanism Using SEM Analysis

The bacterial cells that were determined by the antibiofilm tests to be sensitive were cleaned with PBS three times and fixed using 3.5% glutaraldehyde solution. The microbial cells were repeatedly rinsed with PBS and dried using different concentrations of ethyl alcohol: 30%, 50%, 70%, 90%, and 100% for 15 min at 27 ± 2 °C. The prepared samples were then fixed on an aluminium substrate for SEM/EDX analysis. The morphological features of the control and the ZnCe_x_Fe_2−x_O_4_ nanoparticles (X = 0.06)-treated bacteria were examined using SEM.

### 3.6. Effect of the Synthesised Nanocomposites on Protein Leakage from Bacterial Cell Membranes

To confirm the SEM reaction mechanism of the synthesised nanocomposites against the microbial cell, the protein leakage assay has been conducted [145]. Pure 18 hr bacterial culture was set at 0.5 McFarland (1–3 × 10^8^ CFU/mL), and 100 µL was injected into 10 mL of the nutrient broth, including well-sonicated and dispersed ZnCe_x_Fe_2−x_O_4_ nanoparticles (X = 0, and 0.06) at various concentrations (0.125, 0.25, 0.5, and 1.0 mg/mL). Nanocomposites-free broth injected with culture has been used as the control. All the treated samples were incubated at 37 °C for 5 h and then centrifuged at 15 min at 5500 rpm [146]. For the different samples, 100 μL supernatant was combined with 1 mL of Bradford reagent. Optical density was measured at 595 nm for 10 min of dark incubation [146].

### 3.7. Cell Lines, Treatment, and Reagents

Human breast cancer (MDA-MB-231; triple negative basal B subtype), colon cancer (HT-29; colorectal adenocarcinoma), and Vero kidney epithelial (normal epithelial cells derived from the African green monkey) cell lines provided via the American Type Culture Collection (Rockville, MD, USA) were bought from the Tissue Culture Unit in the Holding Company for Biological Products and Vaccines (VACSERA-Giza, Egypt). Ebselen (Eb) was purchased from Sigma-Aldrich (St. Louis, MO, USA). For western blot analysis, antibodies against Beclin-1 (CAT# ab137161), LC3B (CAT#192890), and P62 (CAT# ab91526) were obtained from Abcam. t-STAT-1 (rabbit polyclonal antibody, PA1-41383), p-STAT-1^(Tyr701)^ (rabbit polyclonal antibody, CAT# 44-376G), t-STAT-6 (mouse monoclonal antibody, CAT#MA5-15659), p-STAT-6^(Tyr641)^ (mouse monoclonal antibody, CAT#700247), t-ERK1/2 (mouse monoclonal antibody, CAT# 14-9108-82), p-ERK1/2^(Thr202,Tyr204)^ (mouse monoclonal antibody, CAT# 14-9109-82), t-JNK (rabbit polyclonal antibody, CAT#51151-1-AP), p-JNK^(Thr183,Tyr185)^ (mouse monoclonal antibody, CAT#MA5-15228), and PARP-1 (mouse monoclonal antibody, CAT#436400) were obtained from ThermoFisher Scientific. ELISA kits for the determination of INF-γ (CAT# MBS824507), TNF-α (CAT# MBS175820), IL-4 (CAT# MBS268288), MDA (CAT# MBS728071), GSH (CAT# MBS042904), GPX (CAT# MBS284182), SOD (CAT# MBS005068), and CAT (CAT#MBS165657) were purchased from MyBioSource (San Diego, CA, USA). ELISA kits for assaying IL-10 (CAT# ab100549), human active caspase-3 (Ser29) (CAT# ab181418), BCL-2 (CAT# ab119506), HIF-1α (CAT# ab82832), and NRF-2 (CAT# ab207223) were obtained from Abcam, and NF-κB (CAT# 85-86081-11) was provided by ThermoFisher Scientific. The other chemicals and reagents used in this study were purchased from Sigma-Aldrich (St Louis, MO, USA).

### 3.8. Culture Media

The cell lines MDA-MB-231, HT-29, and Vero were maintained in DMEM media supplemented with streptomycin (100 mg/mL), penicillin (100 units/mL), and heat-inactivated fetal bovine serum (10%) in a humidity of CO_2_ (5% *v*/*v*) and a temperature of 37 °C.

### 3.9. Subculture of Cell Lines

The cultures were viewed under an inverted microscope (CKX41; Olympus, Japan), to estimate the degree of confluence and to verify the lack of bacterial and fungal contaminants. Cells of MDA-MB-231, HT-29, and Vero were washed with PBS free of Ca^2+^/Mg^2+^, with a volume equivalent to half of the volume of the culture medium. Trypsin/EDTA was then added at 1 mL/25 cm^2^ of surface area, and the flask rotated to merge the trypsin/EDTA with the monolayer. The flasks were incubated for 10 min. Finally, an inverted microscope was used to confirm that the cells had detached.

### 3.10. Sulforhodamine B (SRB) Assay

The cytotoxicity screening of Eb at various concentrations ranging from 0–100 μM and Ce nanoparticles at 10 and 100 μM Ce^3+^ ranging from 0–0.08 in ZnCe_x_Fe_2−x_O_4_ nanoparticles individually on the MDA-MB-231 and HT-29 cell lines was assessed using SRB assays as described by Vichai and Kirtikara [147]. In a 96-well plate, aliquots of 100 μL of cell suspension (5 × 10^3^ cells) were incubated in complete media for 24 h. Then, the cells were treated with another aliquot of 100 μL of medium containing the Eb and Ce nanoparticles at different concentrations. After 72 h of exposure, cells were fixed by replacing the media with 150 μL of 10% TCA and incubating them at 4 °C for 1 h. The TCA solution was removed, and the cells were washed five times with distilled water. Aliquots of 70 μL SRB solution (0.4% *w*/*v*) were added, and the cells were incubated in the dark at room temperature for 10 min. Plates were washed three times with 1% acetic acid and allowed to air-dry overnight. Then, 150 μL of TRIS (10 mM) was added to dissolve the protein-bound SRB stain. The absorbance was measured at 540 nm using a BMG LABTECH^®^-FLUOstar Omega microplate reader. The half-maximal inhibitory concentration (IC_50_) of Eb or Ce in ZnCe_x_Fe_2−x_O_4_ nanoparticles was investigated individually in the MDA-MB-231 and HT-29 cell lines. Then, the Eb and Ce in ZnCe_x_Fe_2−x_O_4_ nanoparticles were used in conjunction to examine their cytotoxicity profiles on the MDA-MB-231 and HT-29 cell lines (based on the IC_50_ identified for each one individually previously) to obtain the final IC_50_ of each nanocomposite on the MDA-MB-231 and HT-29 cell lines. Afterwards, these two final IC_50_ were examined for their cytotoxicity profiles on Vero cells.

### 3.11. Irradiation

The cultured MDA-MB-231 and HT-29 cells were irradiated with a Canadian gamma cell-40 exactor, (^137^Cs) (Best Theratronics Gamma cell 40 Exactor, Ottawa, ON, Canada) at the NCRRT (Cairo, Egypt) at a dose of 4 Gy, with a dose rate of 0.427 Gy/min.

### 3.12. Culture Models and Experimental Protocol

In the current study, the cultures of MDA-MB-231 and HT-29 cells were divided into two sets, as follows:

1. The MDA-MB-231 cell line was divided into four groups. The MDA group: untreated MDA-MB-231 cells line served as control; the MDA + Eb-ZFO group: MDA-MB-231 cells treated with Ebselen (Eb) and ZnFe_2_O_4_ nanoparticles (ZFO); the MDA + IR group: MDA-MB-231 cells exposed to ionising radiation; and the MDA + Eb-ZFO + IR group: MDA-MB-231 cells treated with Eb-ZFO and exposed to IR.
2. The HT-29 cell line was divided into four groups. The HT-29 group: untreated HT-29 cells served as a control; the HT-29 + Eb-ZCFO group: HT-29 cells treated with Eb and ZnCe_0.06_Fe_1.94_O_4_ nanoparticles (ZCFO); the HT-29 + IR group: HT-29 cells exposed to ionising radiation; and the HT-29 + Eb-ZCFO + IR group: HT-29 cells treated with Eb-ZCFO and exposed to IR.

### 3.13. Cell-Cycle Analysis and Apoptosis Detection Using Flow Cytometry

MDA-MB-231 and HT-29 cells were stained with propidium iodide (PI) (Sigma-Aldrich) for cell-cycle analysis, or with PI and annexin V-FITC (BD Biosciences) for the detection of apoptosis. The distribution of cells in the different phases of the cell cycle, based on the differences in DNA content, and the apoptosis positive cells were determined using flow cytometry with a FACS Calibur flow cytometer (BD Biosciences). MDA-MB-231 and HT-29 cells were seeded at a density of 5 × 10^5^/mL in six-well tissue culture plates. After the cells adhered, the MDA-MB-231 cells were treated with Eb-ZnFe_2_O_4_, and the HT-29 cells were treated with ZnCe_0.06_Fe_1.94_O_4_ at the concentrations determined from theIC_50_ obtained from the cytotoxicity screening. Cells were exposed to 4Gy irradiation (either with or without pre-treatment) and cultured for 24 h. The cells were harvested by trypsinisation, washed with PBS, and fixed with pre-chilled 70% ethyl alcohol at 4 °C overnight. The cells were then again washed with PBS and incubated with RNase A for 30 min, followed by staining with 400 μL PI (50 µg/mL PI, 0.1% Triton X-100, and 0.1% sodium citrate in PBS) for 30 min at room temperature in the dark. The percentage of cells in each phase of the cell cycle was calculated using an Accuri C6Flow Cytometer (BD Biosciences, Mountain View, CA, USA). For the detection of apoptosis and necrosis, the cells were harvested using trypsinisation, washed with PBS, and resuspended in 0.5 mL of binding buffer containing 0.5 µg/mL Annexin-V-FITC and 5 µg/mL PI for 30 min in the dark, according to the protocol supplied with the FITC Annexin V apoptosis detection kit (CAT# ab139418, Abcam) and the percent of apoptotic and necrotic cells was assessed using a BD Flow Cytometer (BD Biosciences, USA).

### 3.14. Determination of Intracellular Hydrogen Peroxide (H_2_O_2_)

The concentration of intracellular H_2_O_2_ was measured using colorimetric assay kits (ICT Technologies, CAT#9132).

### 3.15. Quantification of Pro- and Anti-Inflammatory Cytokines, Pro- and Anti-Apoptotic, and Oxidative Stress Markers by ELISA

Levels of INF-γ, TNF-α, IL-4, IL-10, HIF-1, Caspase-3, BCL-2, NF-κB NRF-2, MDA, GSH, GPX, SOD, and CAT were determined according to the protocols accompanying the ELISA kits.

### 3.16. Western Blot Analysis

In a cold homogeniser tube, MDA-MB-231 and HT-29 cells lines were homogenised using a homogenisation lysis buffer (Sigma–Aldrich, St. Louis, MD, USA) according to the method published by [148]. The lysate was centrifuged at 8678× *g* for 20 min at −4 °C, and the protein concentration was measured using BCA protein kits (Thermo Fisher Scientific). Protein aliquots of 7.5 μg from each sample were denatured and loaded onto 8% sodium dodecyl sulphate-polyacrylamide gel electrophoresis, (SDS-PAGE) and transferred to a nitrocellulose membrane (Amersham Bioscience, Piscataway, NJ, USA) using a semidry transfer apparatus (Bio-Rad, Hercules, CA, USA). The membranes were then incubated at 4 °C with 5% non-fat milk blocking buffer, which consisted of Tris–HCl (10 mmol/L^–1^—pH 7.4), Tris-buffered saline with Tween-20 (TBST) (0.05%), and NaCl (150 mmol/L^−1^). The membranes were then washed with TBST and incubated overnight with a 1:1000 dilution of Beclin-1, LC3B II, P62, total and phosphorylated STAT-1 and STAT-6, ERK1/2, JNK, and PARP on a roller shaker at 4 °C. Immunoblotting was performed using the indicated primary antibody and the appropriate horseradish peroxidase (HRP), which was conjugated with goat immunoglobulin (Amersham Biosciences, Piscataway, NJ, USA). Using Amersham^®^ detection kits, chemiluminescence detection was performed according to the manufacturer’s protocols and exposed to X-ray film. The protein levels obtained were estimated using β-actin as a housekeeping arbitrary unit [149].

### 3.17. Statistics

In the analysis of antimicrobial and antibiofilm data, the results were analysed using the least significant difference (LSD) and one-way analysis of variance (ANOVA) followed by Duncan’s multiple range using SPSS version 15. In the analysis of anticancer data, the data were analysed using ANOVA followed by Tukey multiple comparison. Kolmogorov–Smirnov and Bartlett’s tests were used to evaluate the normality of the distribution and the homology of the variance, respectively. Statistical analyses were performed using GraphPad Prism, Version 6 (GraphPad Software, San Diego, CA, USA, www.graphpad.com). All tests were two-tailed, and *p*-values < 0.05 were considered to be statistically significant.

## 4. Conclusions

A cost-effective sol–gel method was used to prepare ZFO or ZCFO ferrites, which were later conjugated with Eb. The antimicrobial and antibiofilm were proved against a wide array of pathogens. Moreover, the antiproliferative, antioxidant, and anti-inflammatory abilities of the prepared systems against human breast cancer (MDA-MB-231) and colon cancer (HT-29) cell lines were studied in detail. XRD analysis confirmed the chemical structure and crystallinity of the prepared samples, which possessed Fd3 m space groups. The chemical composition and purity of the materials were confirmed by EDX and FTIR analyses. Finally, the external morphology and the porous nature of the prepared samples were investigated using SEM analysis. The antimicrobial activity of the synthesised ZnCe_x_Fe_2−x_O_4_ increased as the x value increased. The highest ZOI of *S. aureus* was observed when X = 0.06 in ZnCe_x_Fe_2−x_O_4_ and slightly decreased when X = 0.08. In the case of *C. albicans*, the active sample was ZnCe_x_Fe_2−x_O_4_ with X =0.06. The semi-quantitative determination of the inhibition percentage was estimated following the elimination of CV-stained biofilms, and the results showed the percentage of inhibition for *S. aureus* (92.73%), *P. mirabilis* (79.54%), and *C. albicans* (90.18%) following the addition of 10.0 µg/mL ZnCe_x_Fe_2−x_O_4_; X = 0.06.After treatment with ZnCe_x_Fe_2−x_O_4_; X = 0.06, morphological differences in *S.* *aureus* were apparent, and a complete lysis of bacterial cells, with a concomitant decrease in the viable number, and ultimately the growth of biofilm was inhibited. These results reflected the antimicrobial activity of Ce addition in the synthesised ZnCe_x_Fe_2−x_O_4_; X = 0.06. Overall, we conclude that Eb-ZFO or Eb-ZCFO with or without IR affected the fine-tuning of intracellular ROS signalling in MDA-MB-231 and HT-29 cells by reducing ROS production through altering the antioxidant defence machinery associated with the deactivation of the ERK1/2, JNK, NRF-2, TNF-α/NF-κB, INF-γ/STAT-1, and IL-4/STAT-6 signalling pathways. The mechanism of Eb-ZFO in the MDA-MB-231 cell line depends primarily on the Eb antioxidant/anti-inflammatory capability and the cytotoxicity profile of the ZnO-NPs. However, the antitumour activity of Eb-ZCFO in the HT-29 cell line depends on Ce nanoparticles as ROS scavengers and Eb as an immune modulator of the TME.

## Figures and Tables

**Table 1:** Absorption bands at the tetrahedral and octahedral sites of ZnCe_x_Fe_2−x_O_4_ (X = 0.00, 0.02, 0.04, 0.06, and 0.08).

		υ_1_	υ_2_
Doped Ferrites	*x*	cm^−1^	cm^−1^
**ZnCe_x_Fe_2−x_O_4_**	0.00	594.75	404.81
	0.02	549.12	429.17
	0.04	547.97	427.66
	0.06	553.24	426.81
	0.08	549.99	400.32

**Table 2:** Antibacterial and antifungal activities of ZnCe_x_Fe_2−x_O_4_ against some multi-drug resistant bacteria and pathogenic *Candida* species according to ZOI (mm) and MIC (μg/mL).

Pathogenic Microbes	ZOI (mm), ZnCe_x_Fe_2−x_O_4_ (10 µg/mL)					ZnCe_x_Fe_2−x_O_4_; X = 0.06 (Starting with 50 µg/mL Concentration)	AX & NS
	X = 0	X = 0.02	X = 0.04	X = 0.06	X = 0.08		
*Staphylococcus aureus*	Nil	Nil	9.0 ^c^ ± 0.6545	13.2 ^d^ ± 0.2335	10.1 ^c^ ± 0.2335	0.390	Nil
*Pseudomonas aeruginosa*	Nil	7.0 ^a^ ± 0.5000	9.0 ^c^ ± 0.5000	9.5 ^bc^ ± 0.5000	9.5 ^bc^ ± 0.555	6.250	Nil
*Escherichia coli*	7.0 ^a^ ± 0.5755	Nil	7.0 ^a^ ± 1.0000	8.5 ^a^ ± 0.6545	8.0 ^a^ ± 0.6545	12.50	Nil
*Klebsiella pneumoniae*	Nil	Nil	Nil	8.0 ^a^ ± 0.5755	Nil ± 0.6387	6.250	Nil
*Proteus vulgaris*	Nil	Nil	8.0 ^b^ ± 0.5755	9.0 ^b^ ± 0.2335	8.5 ^ab^ ± 0.2335	12.50	Nil
*Salmonella typhi*	Nil	Nil	Nil	9.0 ^b^ ± 0.6545	Nil	12.50	Nil
*Proteus mirabilis*	Nil	Nil	Nil	9.5 ^c^ ± 1.0000	8.5 ^b^ ± 0.2335	6.250	Nil
*Candida albicans*	Nil	Nil	9.5 ^d^ ± 0.6387	13.5 ^d^ ± 0.5000	10.9 ^c^ ± 0.2335	0.195	Nil
*Candida tropicalis*	Nil	Nil	Nil	8.9 ^ab^ ± 0.6545	8.0 ^a^ ± 0.6387	12.50	Nil
LSD	---------	---------	0.33330	1.00500	0.12552	--------	-------

Values are means ± SD (n = 3). Data within the groups were analysed using one-way ANOVA followed by ^a,b,c,d^ Duncan’s multiple range test (DMRT) and LSD = least significant differences. Nil means that no ZOI had been measured and therefore no activity of the tested samples. AX = Amoxicillin (antibacterial standard). NS = Nystatin (antifungal standard).

**Table 3:** Semi-quantitative inhibition of biofilm formation for bacterial and yeast pathogens non-treated and treated with ZnCe_x_Fe_2−x_O_4_; X = 0.06.

Bacterial and Yeast Strains	O.D. of Crystal Violet Stain at 570.0 nm		Inhibition %
	Control	ZnCe_x_Fe_2−x_O_4_; X = 0.06 (10.0 µg/mL)	
*Staphylococcus aureus*	1.058 ^e^ ± 0.0080	0.020 ^a^ ± 0.0021	92.73%
*Pseudomonas aeruginosa*	0.945 ^d^ ± 0.0062	0.241 ^c^ ± 0.0047	78.83%
*Escherichia coli*	0.877 ^b^ ± 0.0070	0.298 ^c^ ± 0.0053	75.27%
*Klebsiella pneumoniae*	0.998 ^d^ ± 0.0025	0.388 ^d^ ± 0.0062	61.24%
*Proteus vulgaris*	0.899 ^c^ ± 0.0046	0.444 ^e^ ± 0.0036	56.29%
*Salmonella typhi*	1.222 ^f^ ± 0.0070	0.831 ^a^ ± 0.0053	26.18%
*Proteus mirabilis*	0.989 ^d^ ± 0.0062	0.211 ^c^ ± 0.0047	79.54%
*Candida albicans*	0.999 ^d^ ± 0.0080	0.099 ^b^ ± 0.0021	90.18%
*Candida tropicalis*	0.557 ^a^ ± 0.0080	0.451 ^e^ ± 0.0021	34.16%
LSD	0.01767	0.01267	------------

Values are means ± SD (n = 3). Data within the groups were analysed using one-way analysis of variance (ANOVA) followed by ^a,b,c,d,e,f^ Duncan’s multiple range test (DMRT) and LSD = least significant difference.

## Data Availability

Not applicable.

## Supplementary Materials

Supplementary Materials are available online at https://www.mdpi.com/article/10.3390/ijms221810171/s1.
