# Supplementary material for: Influence of Ce3+ Substitution on Antimicrobial and Antibiofilm Properties of ZnCexFe2−xO4 Nanoparticles (X = 0.0, 0.02, 0.04, 0.06, and 0.08) Conjugated with Ebselen and Its Role Subsidised with γ-Radiation in Mitigating Human TNBC and Colorectal Adenocarcinoma Proliferation In Vitro
Source: Int J Mol Sci. 2021 Sep 21;22(18):10171. doi: 10.3390/ijms221810171 (PMC8466506; doi:10.3390/ijms221810171)
Supplement: Supplementary file 1 [file ijms-22-10171-s001.zip › ijms-1395458-supplementary.pdf]

I) MDA-MB-231

a) Eb

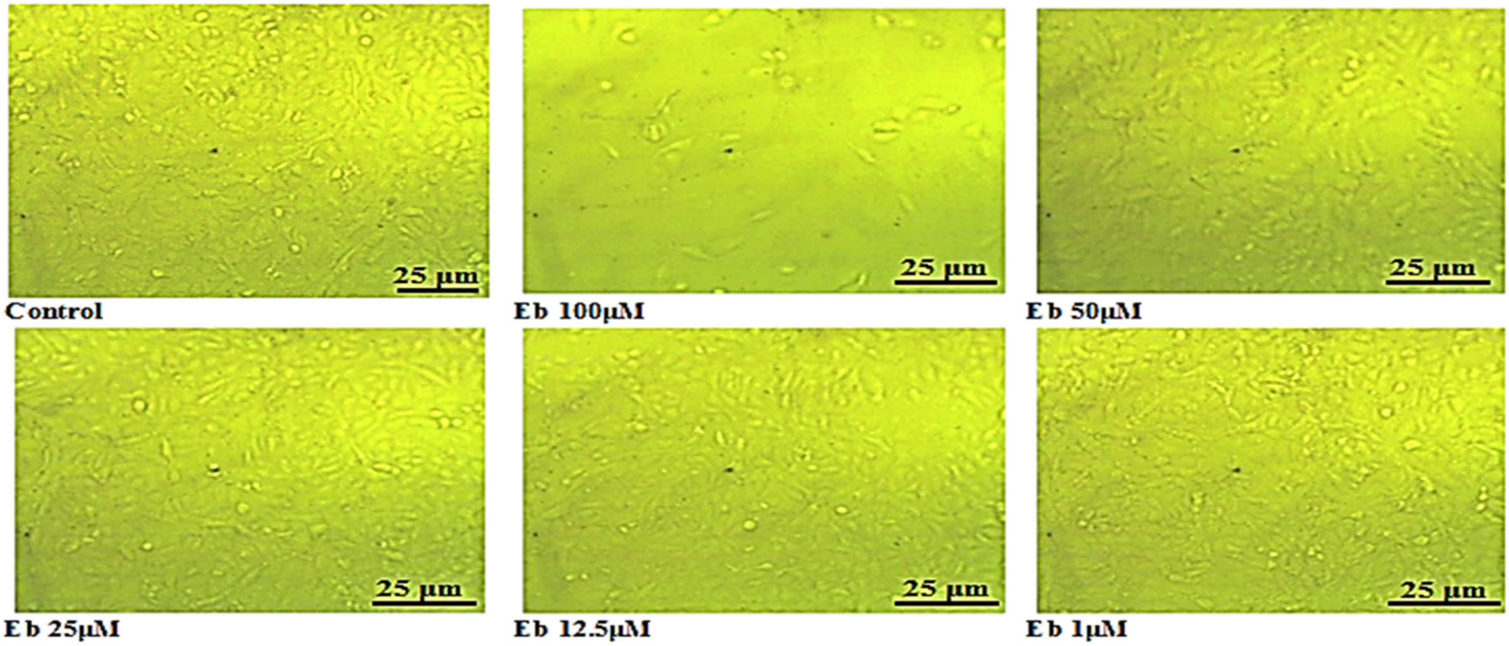

b) $ZnCe_xFe_{2-X}O_4$

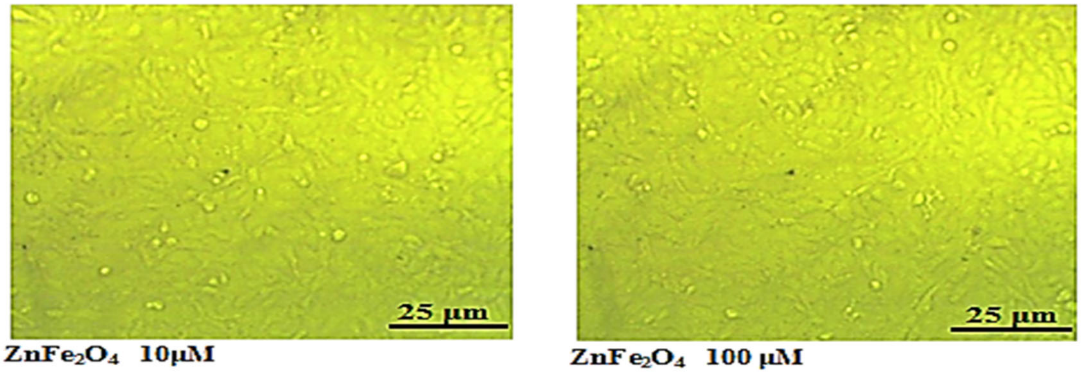

c) Eb-$ZnFe_2O_4$

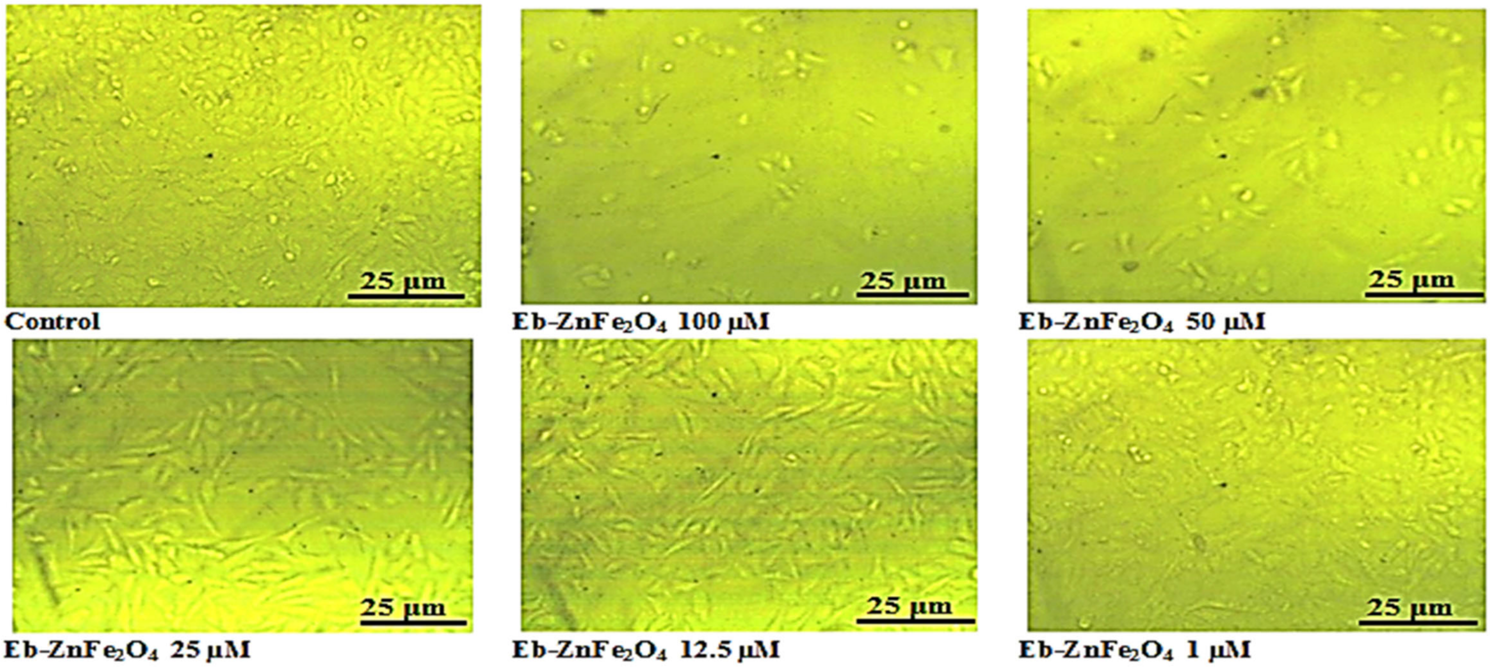

II) HT-29 cells

a) Eb

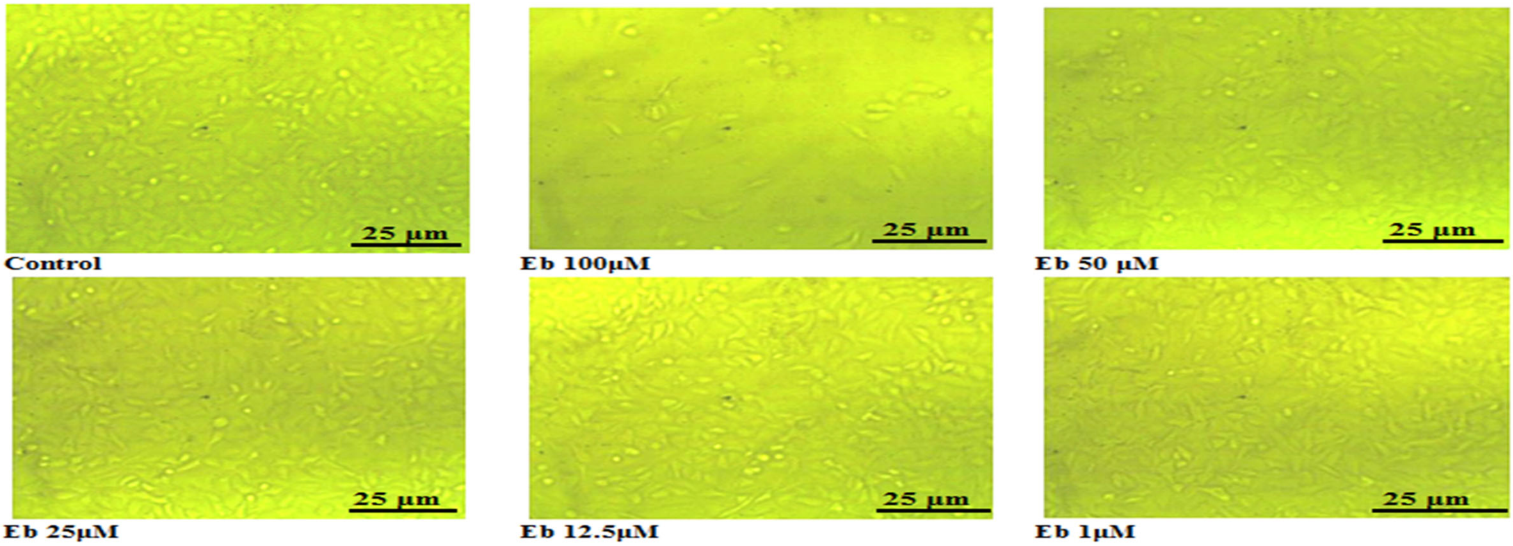

b) $ZnCe_xFe_{2-X}O_4$

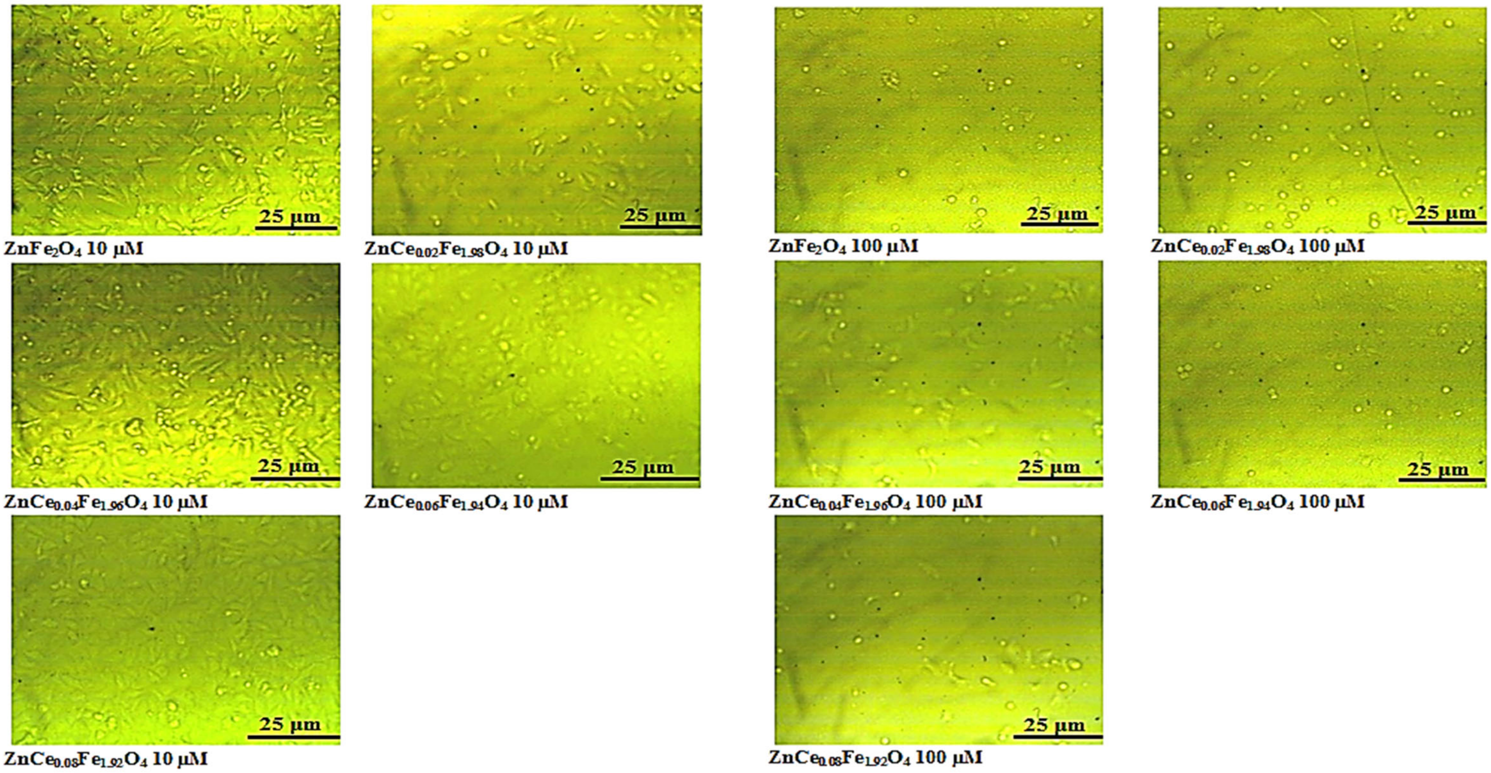

c) Eb-$ZnCe_{0.06}Fe_{1.94}O_4$

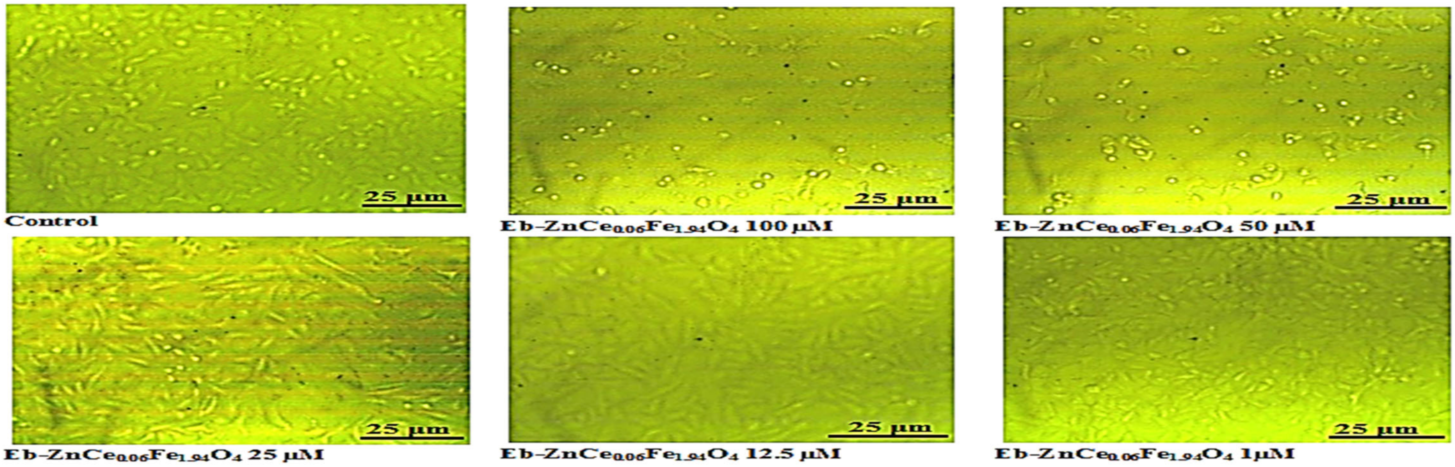

III) Normal Vero cells

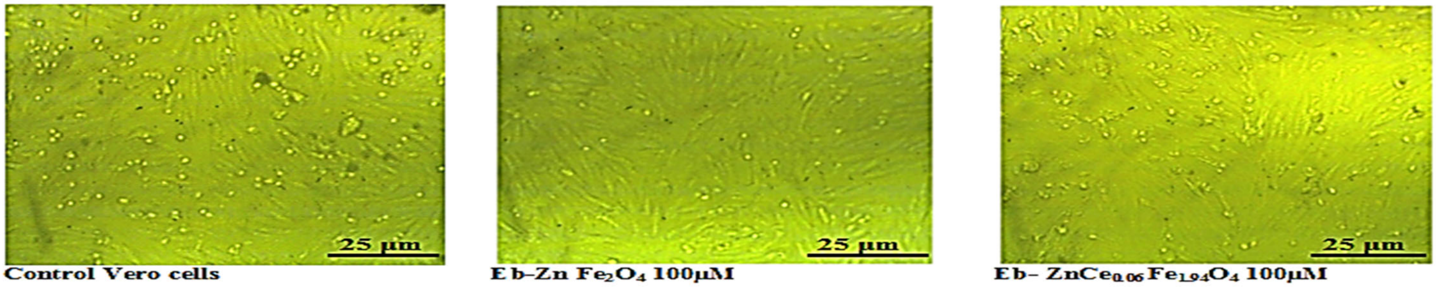

Supplement Figure S1: Inverted light microscopy images of cytotoxicity screening of the various concentrations of Ebselen (Eb) and/or $ZnCe_xFe_{2-X}O_4$ nanoparticles. I) MDA-MB-231 cells were treated as follows: a) Eb, b) $ZnCe_xFe_{2-X}O_4$ (here we show only the images of the low and high concentrations of the effective nanoparticles, due to the other nanoparticles were not effective with no apparent cytotoxicity and were quietly as same as the control) and c) Eb-$ZnFe_2O_4$. II) HT-29 cells were treated as follows: a) Eb, b) $ZnCe_xFe_{2-X}O_4$ and c) Eb-$ZnCe_{0.06}Fe_{1.94}O_4$. III) Normal Vero cells treated by Eb-$ZnFe_2O_4$ and Eb-$ZnCe_{0.06}Fe_{1.94}O_4$ at concentration of 100 µM.
